# Multiplex MinION sequencing suggests enteric adenovirus F41 genetic diversity comparable to pre-COVID-19 era

**DOI:** 10.1099/mgen.0.000920

**Published:** 2023-01-06

**Authors:** Mailis Maes, Fahad Khokhar, Sam A. J. Wilkinson, Andrew D. Smith, Ganna Kovalenko, Gordon Dougan, Joshua Quick, Nicholas J. Loman, Stephen Baker, Martin D. Curran, Jordan P. Skittrall, Charlotte J. Houldcroft

**Affiliations:** ^1^​ Clinical Microbiology and Public Health Laboratory, UK Health Security Agency, Addenbrooke’s Hospital, Cambridge, UK; ^2^​ Cambridge Institute of Therapeutic Immunology and Infectious Disease, School of Clinical Medicine, University of Cambridge, Cambridge Biomedical Campus, Cambridge CB2 0AW, UK; ^3^​ Institute of Microbiology and Infection, School of Biosciences, University of Birmingham, Birmingham, UK; ^4^​ Division of Virology, Department of Pathology, University of Cambridge, Cambridge, UK; ^5^​ Department of Medicine, School of Clinical Medicine, University of Cambridge, Cambridge Biomedical Campus, Cambridge, UK; ^6^​ Cambridge University Hospitals NHS Foundation Trust, Cambridge, UK; ^7^​ Department of Genetics, University of Cambridge, Cambridge, UK

**Keywords:** adenovirus, DNA virus, genetic epidemiology, genomics, hepatitis, virology

## Abstract

Human adenovirus F41 causes acute gastroenteritis in children, and has recently been associated with an apparent increase in paediatric hepatitis of unknown aetiology in the UK, with further cases reported in multiple countries. Relatively little is known about the genetic diversity of adenovirus F41 in UK children; and it is unclear what, if any, impact the COVID-19 pandemic has had on viral diversity in the UK. Methods that allow F41 to be sequenced from clinical samples without the need for viral culture are required to provide the genomic data to address these questions. Therefore, we evaluated an overlapping-amplicon method of sequencing adenovirus genomes from clinical samples using Oxford Nanopore technology. We applied this method to a small sample of adenovirus-species-F-positive extracts collected as part of standard care in the East of England region in January–May 2022. This method produced genomes with >75 % coverage in 13/22 samples and >50 % coverage in 19/22 samples. We identified two F41 lineages present in paediatric patients in the East of England in 2022. Where F41 genomes from paediatric hepatitis cases were available (*n*=2), these genomes fell within the diversity of F41 from the UK and continental Europe sequenced before and after the 2020–2021 phase of the COVID-19 pandemic. Our analyses suggest that overlapping amplicon sequencing is an appropriate method for generating F41 genomic data from high-virus-load clinical samples, and currently circulating F41 viral lineages were present in the UK and Europe before the COVID-19 pandemic.

## Data Summary

All read data have been deposited in the European Nucleotide Archive (ENA), under accession numbers ERR9939847–ERR9939870. A Microreact project associated with this study is available: https://microreact.org/project/bYhycuN63ZToY7348qJHbr-adenoviruscambridge.

**Impact Statement**

Since January 2022, human adenovirus F41 (HAdV-F41) has been associated with an increase in cases of paediatric hepatitis of unknown aetiology. To test hypotheses about the relationship between this DNA virus that normally causes acute gastroenteritis and a novel disease presentation, it is necessary to compare HAdV-F41 sequences from paediatric hepatitis cases and cases with typical HAdV-F41 symptoms. We use a novel method for recovering HAdV-F41 genomes from clinical samples using overlapping PCR and Nanopore sequencing, and discuss future improvements in coverage of hypervariable regions. This study provides, to our knowledge, the first publicly available HAdV-F41 sequence data from 21 children with adenovirus gastroenteritis, providing important context for studies of viral variants associated with novel disease presentations. Finally, we show that available HAdV-F41 genomes from paediatric hepatitis cases fall into viral lineages that have been circulating in the UK and Germany since before the introduction of COVID-19 restrictions in 2020–2021, and the emergence of a new viral lineage with altered tissue tropism in 2022 is unlikely to explain the newly identified association between paediatric hepatitis and HAdV-F41. This study will be of interest to those taking genomic surveillance technologies from SARS-CoV-2 (severe acute respiratory syndrome coronavirus 2) and applying them to more ancient and genetically diverse human pathogens.

## Introduction

Human adenoviruses (HAdVs) (genus *Mastadenovirus*) are non-enveloped dsDNA viruses with genomes approximately 35 kb in length. Since their identification in the 1950s [1, 2], seven species (A–G) and over 100 types have been identified as associated with disease, including acute or persistent infections, and symptoms ranging from conjunctivitis and upper-respiratory tract disease to acute gastroenteritis (AGE). In the immunocompromised host, a variety of adenovirus types have been associated with disseminated disease [3].

HAdV species F is composed of two types, 40 and 41, both associated with gastrointestinal disease. HAdV-F41 was first identified as a diarrhoeal pathogen in 1973 in The Netherlands [4]. It has been recognized worldwide as a leading causative agent of diarrhoea in children [5, 6] and also as a nosocomial pathogen that may cause outbreaks among immune-compromised individuals, e.g. paediatric haematopoietic stem cell transplant recipients [7]. Because of their shared enteric tropism and genome structure, most studies do not differentiate between infection with HAdV-F40 and -F41. Despite their closely related neutralization profiles, distinguishing these types is possible using mAbs, RFLP assays [8], quantitative PCR (qPCR) and genomic sequencing [9, 10].

There has been an apparent increase in paediatric gastrointestinal adenovirus infections [11, 12] and an increase in reported acute hepatitis cases of unknown aetiology in children (AHUAC) not caused by hepatitis viruses A–E in the UK during 2022 [13], with cases reported from other countries worldwide but predominantly from Europe and the Americas [14–18]. As of 26/08/2022, 273 confirmed cases of AHUAC have been identified in the UK [19] and 65 % of cases tested for adenovirus (blood or stool) were positive [20]. Of the HAdVs that were successfully typed, 77 % were type F41 [11, 12, 21, 22]. The emergence of a new F41 lineage associated with a change in disease presentation or tissue tropism has been hypothesized as one reason for the association of HAdV with AHUAC. Relatively little is known about HAdV-F41 genomic diversity in adults or children from the UK. Some data are available from immune-compromised and hospitalized children from before the COVID-19 pandemic [23, 24], and 2011–2019 and 2021–2022 samples are available from Germany [25]. Therefore, it is important to have access to HAdV-F41 genomes associated with gastrointestinal disease from the UK from the period after 2020–2021 COVID-19 restrictions.

Overlapping PCR amplicon sequencing methods have previously been used for viruses such as Ebola, Zika and SARS-CoV-2 (severe acute respiratory syndrome coronavirus 2) [26–29]. PCR-based amplification removes the need for viral culture, which other studies have used to amplify HAdV-F41 for sequencing [7]. Therefore, we screened samples (adults and children) from the East of England region for the presence of HAdV-F DNA, with the aim of evaluating/assessing the success of an overlapping amplicon sequencing of HAdV-F41, to establish assay performance and to provide data on HAdV-F41 diversity in samples not associated with AHUAC.

## Methods

### Samples

**Fig. 1.**
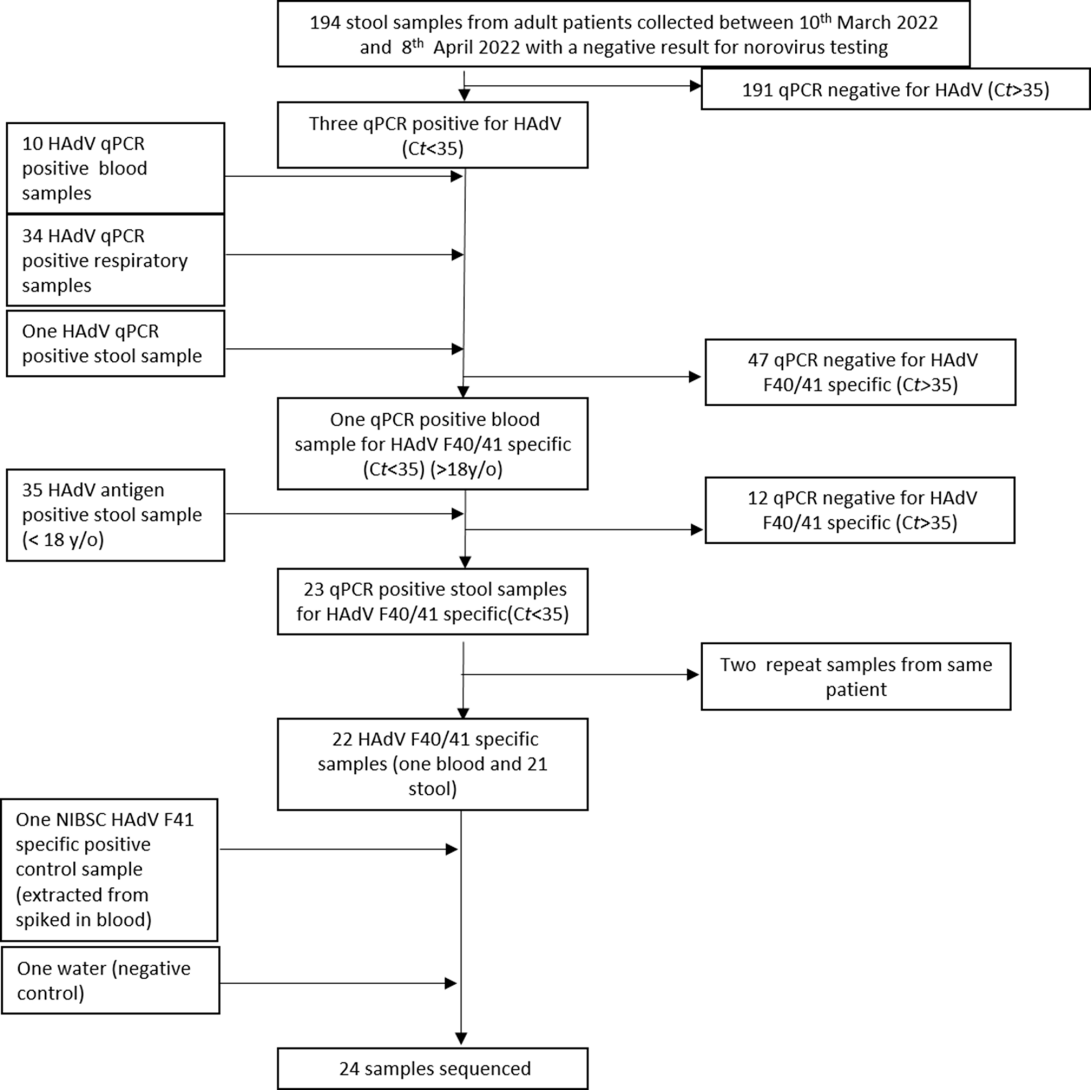
Study flow diagram. Diagram showing the sample identification strategy for HAdV-F positive extracts used in this study. y/o, Years old.

We initially prioritized residual diagnostic stool samples from adults for HAdV-F testing, including individuals with AGE symptoms who tested negative for norovirus, and other HAdV-positive samples identified in routine clinical care (Fig. 1). In order to have sufficient samples suitable for HAdV sequencing, we also included paediatric samples with a positive HAdV-antigen test result. In total, 22 samples were identified that were positive for HAdV species F by qPCR.

### Nucleic acid extractions

Nucleic acid extractions from blood, stool and respiratory (swab) samples were performed on the automated QIAsymphony platform using the virus mini kit (cat. no. 955134), with sample volume set at 1 ml and an elution volume of 110 µl. Samples of 200 µl BAL (bronchoalveolar lavage) and National Institute for Biological Standards and Control (NIBSC) control material were extracted using the Qiagen EZ1 (cat. no. 937036) with an elution volume of 150 µl.

### HAdV-F typing

Samples that were positive by routine qPCR using adenovirus specific primers against the hexon gene [30] (130 bp product) or rapid antigen test (Coris BioConcept) for adenovirus (any type) were screened using a qPCR assay for the presence of adenovirus species F using primers specific for the fibre gene (150 bp product), modified from published work [31] and described in Table 1.

**Table 1.** Primers used in this study.

Type	Target	Name	Sequence (5'→3')	5' mod	3' mod	Final concn (µM)
Forward	Fibre	H-Aden-F	CAC TTA ATG CTG ACA CGG GC*	–	–	0.6
Reverse	Fibre	H-Aden-R	ACT GGA TAG AGC TAG CGG GC*	–	–	0.6
Probe	Fibre	H-Aden-P	T[G]C A[C]C TC[T] TG[G] AC[T] AGT (LNA probe)	Cy5	BHQ2	0.1

*Bases marked in bold represent modifications from published primers [31].

### Sample selection

Samples were received as part of the routine diagnostic service by our laboratory, which provides public-health and regional reference laboratory services to the East of England, as well as routine hospital microbiology services to local teaching and specialist hospitals and nearby district general hospitals. The sample selection strategy (Fig. 1) aimed to find sufficient residual samples with surplus to facilitate our investigation. Initially, stool samples submitted between 10th March and 8th April 2022 from patients over 18 years of age with a negative result from norovirus testing (*n*=194) were tested with a monoplex qPCR for HAdV [30]. Where HAdV was detected, samples were screened by qPCR with HAdV-F40/41 specific primers. Secondly, previously stored qPCR positive HAdV extracts from blood (*n*=10), respiratory (*n*=34) and stool (*n*=1) samples submitted between January and May 2022 from patients of all ages were screened using HAdV-F40/41 specific primers. Finally, stored stool samples with a positive HAdV hexon antigen test (*n*=35) collected between January and May 2022 from under 18 year olds, or adult samples where adenovirus antigen testing was requested as part of clinical care, were extracted using the previously described extraction method and tested by qPCR for HAdV (all types) and HAdV-F40/41.

### Primer design – overlapping amplicons

Whole-genome sequences for 125 HAdV-F isolates over 20 kb in length were downloaded from GenBank. Of these, 113 genomes (112 F41 genomes and the F reference genome NC_001454) were used for primer design (Table S1, available with the online version of this article). A masked reference (variant positions masked with IUPAC ambiguity codes) was generated using NMaskGen_Snakemake (https://github.com/ChrisgKent/NMaskGen_Snakemake). Using this mask, a scheme consisting of 92 1.2 kb overlapping amplicons was designed using a version of the PrimalScheme software modified to output four pools (https://github.com/quick-lab/HAdV/blob/main/HAdV-F41/v1.0/HAdV-F41_1200jh.primer.bed). This so called ‘jackhammer’ approach was added to provide additional redundancy against amplification failures.

### PCR amplification of viral genomes, sequencing and genome assembly

Whole-genome sequencing was attempted on all HAdV-F positive samples with a cycle threshold (*C*_t_) value <35, excluding repeat samples from the same patient. Sequencing was performed at the University of Cambridge/UK Health Security Agency (UKHSA) in May 2022. Three blood DNA extracts that were HAdV negative (qPCR) were used as negative PCR amplification controls for sequencing.

Viral genomes were amplified with a multiplex PCR approach consisting of 1.2 kb overlapping amplicons (Table S2). Sequencing libraries were prepared using a modified ARTIC Lo-Cost sequencing protocol, adapted for four input primer sets (Table S3) and omitting the Reverse transcription (RT)-PCR steps (https://www.protocols.io/view/ncov-2019-sequencing-protocol-v3-locost-bp2l6n26rgqe/v3). A detailed protocol is available in the Supplementary Methods.

Genomes with greater than 25 % missing data (Ns) were excluded from further analysis. Raw read data for all samples were deposited in the European Nucleotide Archive (ENA), under accession numbers ERR9939847– ERR9939870 (Table S4).

### Alignment and phylogenetics

Whole-genome HAdV sequences available in GenBank on 27/06/2022 were downloaded. Sequences [32] were excluded from further analysis if they had large numbers of sequence gaps, in line with published work [25, 33]. One HAdV-F41 genome sequence associated with AHUAC was generated at the Centre for Virus Research, Glasgow, UK [12], and kindly provided by Richard Orton and Emma Thomson. Published genomes were aligned using mafft [34] with default settings and missing bases treated as wildcards. Sequences generated from patient samples were then aligned to sequences from GenBank using the --add and –keep-length options. Alignments were manually corrected (removal of N insertions where no sequence is present in any other F40 or F41 genome) in mega11 [35].

Maximum-likelihood phylogenies were reconstructed using iq-tree v1.6.12 [36, 37] using default settings and 1000 bootstrap replicates. Trees were visualized in iTOL [38]. F41 lineages were assigned based on the clusters and nomenclature from published work [25]. SimPlot++ v1.3 was used to generate similarity plots (SimPlots) using a window size of 500 bp, and a step size of 100 bp, using the Jukes–Cantor model [39].

## Results

### Detection of HAdV antigen in paediatric and adult samples

HAdV-F-positive residual diagnostic samples were identified using three approaches (Fig. 1). All HAdV-positive samples identified were then tested for the presence of HAdV-F40/41 DNA using specific qPCR primers that detect all members of *Mastadenovirus* species F, but do not distinguish between the two types [31].

**Fig. 2.**
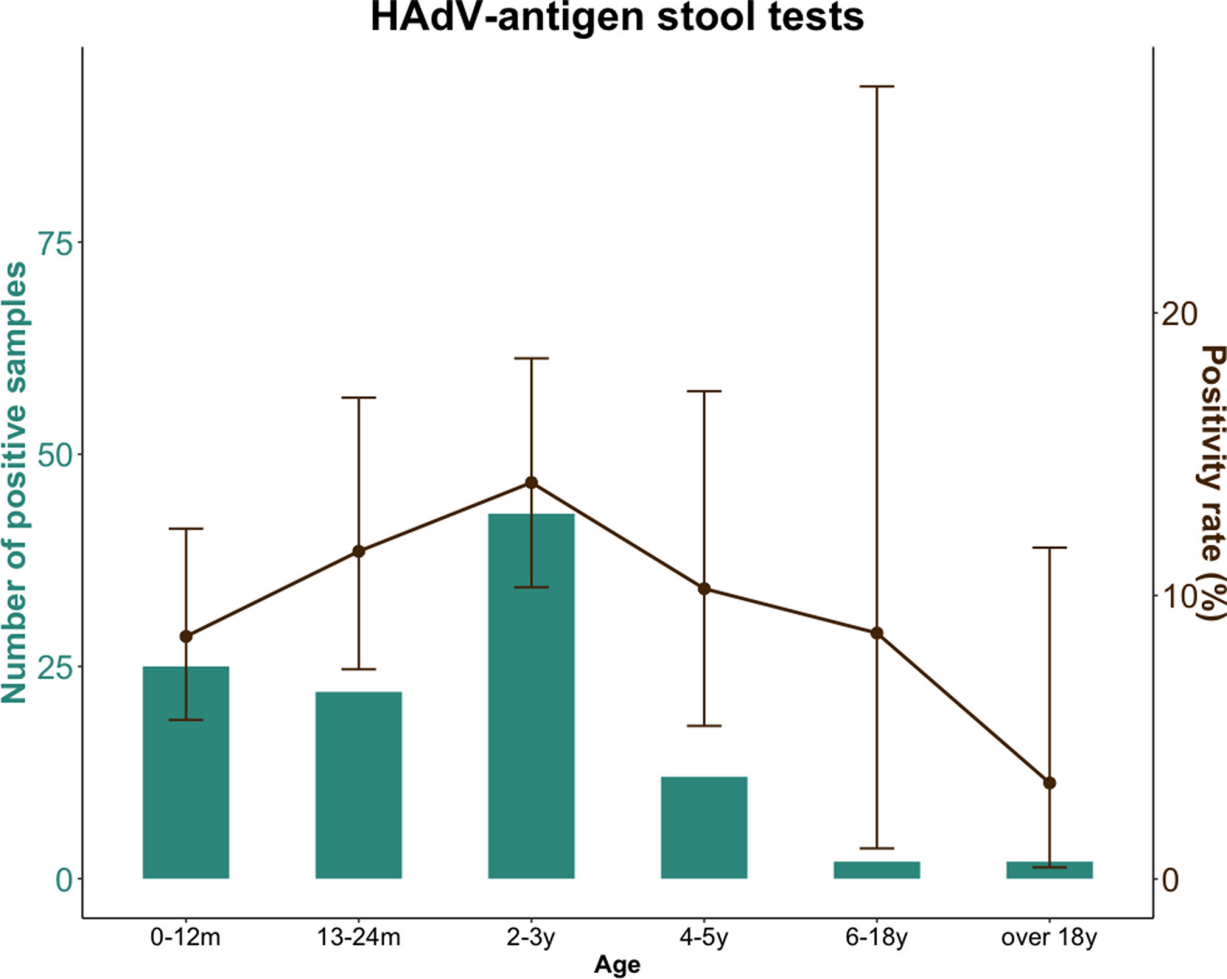
Number of HAdV-antigen-positive stool samples and positivity rate during January–May 2022, by age group (green bars). Brown dots show percentage positivity rate, ranges shown on the positivity rate are 95 % confidence intervals calculated using the Clopper–Pearson method. m, Months; y, years.

Of the 35 available HAdV-antigen-positive stool samples, 23 were HAdV-F40/41 positive (*C*_t_ <16); however, 2 samples were additional samples from a previous sampled patient. During the period January to May, the highest percentage of positive HAdV-antigen tests was reported from the age group 2–3 years (Fig. 2). In total, of all HAdV positive samples (any type): 1/10 blood samples, 0/34 respiratory samples and 23/39 stool samples were positive for HAdV-F40/41 (Fig. 1).

### Patient characteristics

The clinical characteristics of each positive case were reviewed to identify underlying immune suppressive conditions that may have predisposed the individuals to adenovirus infection. The sole HAdV-F-positive test result in an adult (>18 years of age) was an individual with a haematological malignancy. Two of the paediatric patients had haematological conditions associated with immune suppression. One paediatric patient was receiving inpatient care following premature birth, and had previously received treatment for necrotizing enterocolitis. One paediatric patient had a norovirus co-infection. One paediatric patient had a history of recent international travel (discussed below).

### Sequencing HAdV-F41 from clinical samples

One adult blood (HAdV-F40/41 specific *C*_t_=24.6) and 21 paediatric samples (HAdV-F40/41 specific *C*_t_=4.58–15.2) with a positive HAdV-F40/41 qPCR result were available for sequencing. HAdV-F41 control material from NIBSC/UKHSA Colindale (extracted from spiked blood; HAdV-F specific *C*_t_=23.1) was used as the positive control. Molecular grade water was used as the negative control.

**Fig. 3.**
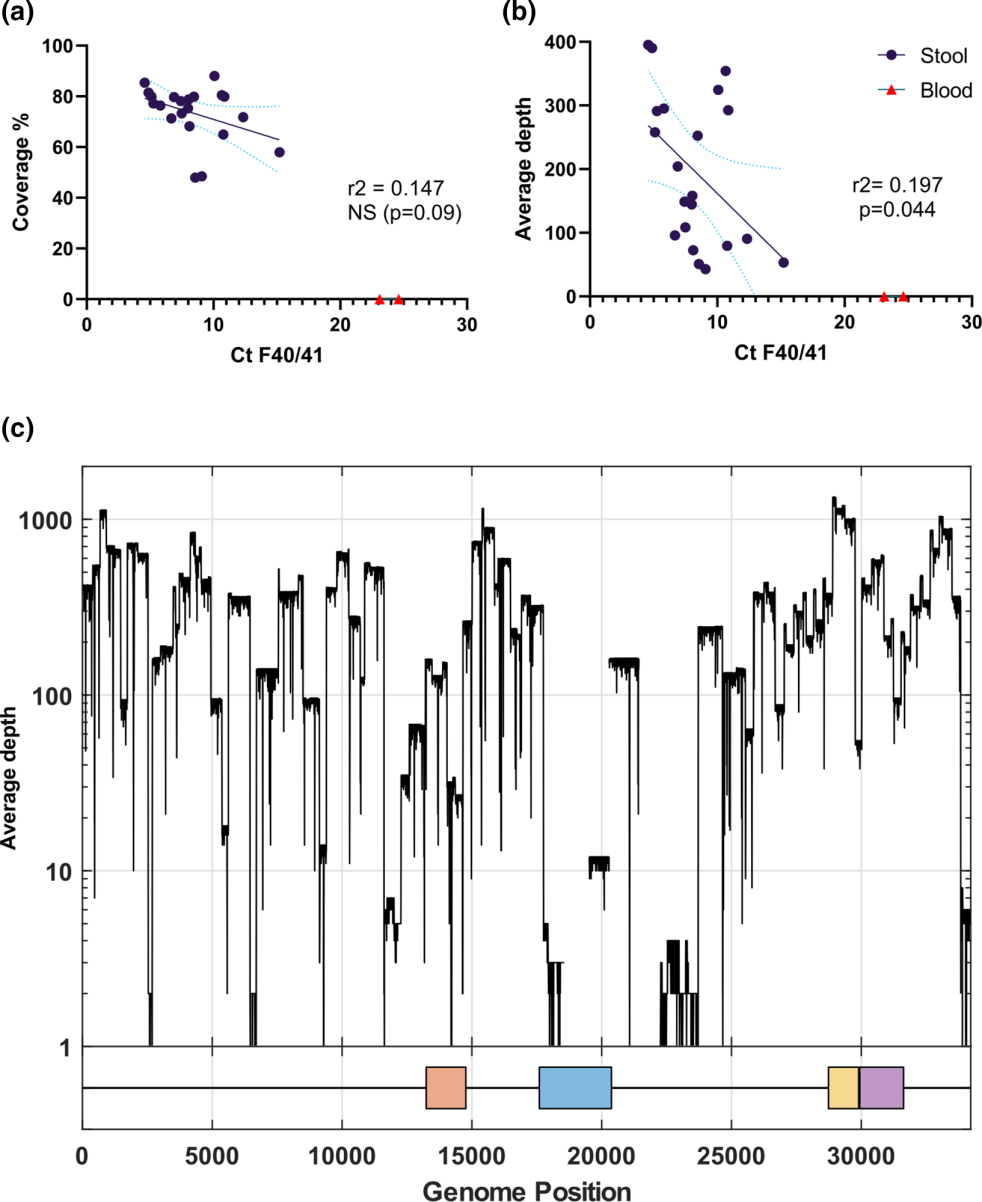
(a, b) Scatter plots showing (**a)** sample *C*_t_ value versus per cent genome coverage; (**b)** sample *C*_t_ value versus mean sequencing depth; in both cases, regressions exclude the samples extracted from blood that did not produce any HAdV-F reads. (**c)** The depth of coverage relative to the adenovirus F RefSeq (NC001454.1) for sample ERR9939847, a HAdV-F41 genome derived from a paediatric stool sample. The four capsid proteins are shown on the *x*-axis: penton (orange), hexon (blue), short fibre (yellow) and long fibre (purple).

There was a no statistically significant relationship (r^2^=0.147, *P*=0.09) between input *C*_t_ value (a proxy for virus load) and sequencing coverage (number of bases with a valid call) when the blood-derived samples were excluded (Fig. 3a). There was a weak relationship between the mean depth of coverage at each base, averaged across the whole genome (r^2^=0.197, *P*=0.044) (Fig. 3b). As there were no samples available with *C*_t_ values >16 that produced HAdV-specific reads, we were not able to ascertain a minimum input *C*_t_ for successful sequencing.

It is also unclear whether the failure to recover HAdV-F41 reads from the adult blood sample and the control material was a result of the sample types. Both samples were qPCR positive on two different assays, although the PCR product size for qPCR is shorter than the product size generated by the ARTIC HAdV-F protocol. Attempts to amplify HAdV PCR products from HAdV qPCR-negative blood DNA extracts did not generate PCR products, with no evidence of PCR mispriming against host DNA (Fig. S1). Increasing the number of cycles of amplification from 25 to 35 did not generate a PCR product in the qPCR-positive blood-derived extracts.

In samples with >75 % genome coverage, the mean depth of coverage ranged from 145× to 395×. In all samples with mapped reads, mean depth of coverage was reduced in the L3 hexon and L4 hexon assembly protein (Fig. 3c). These two regions show the lowest similarity between HAdV-F40 and -F41, but are similar between F41 lineages (lineage 1 shown as the SimPlot query and lineage 3b shown in blue; Fig. S2).

### Phylogenetic analysis

**Fig. 4.**
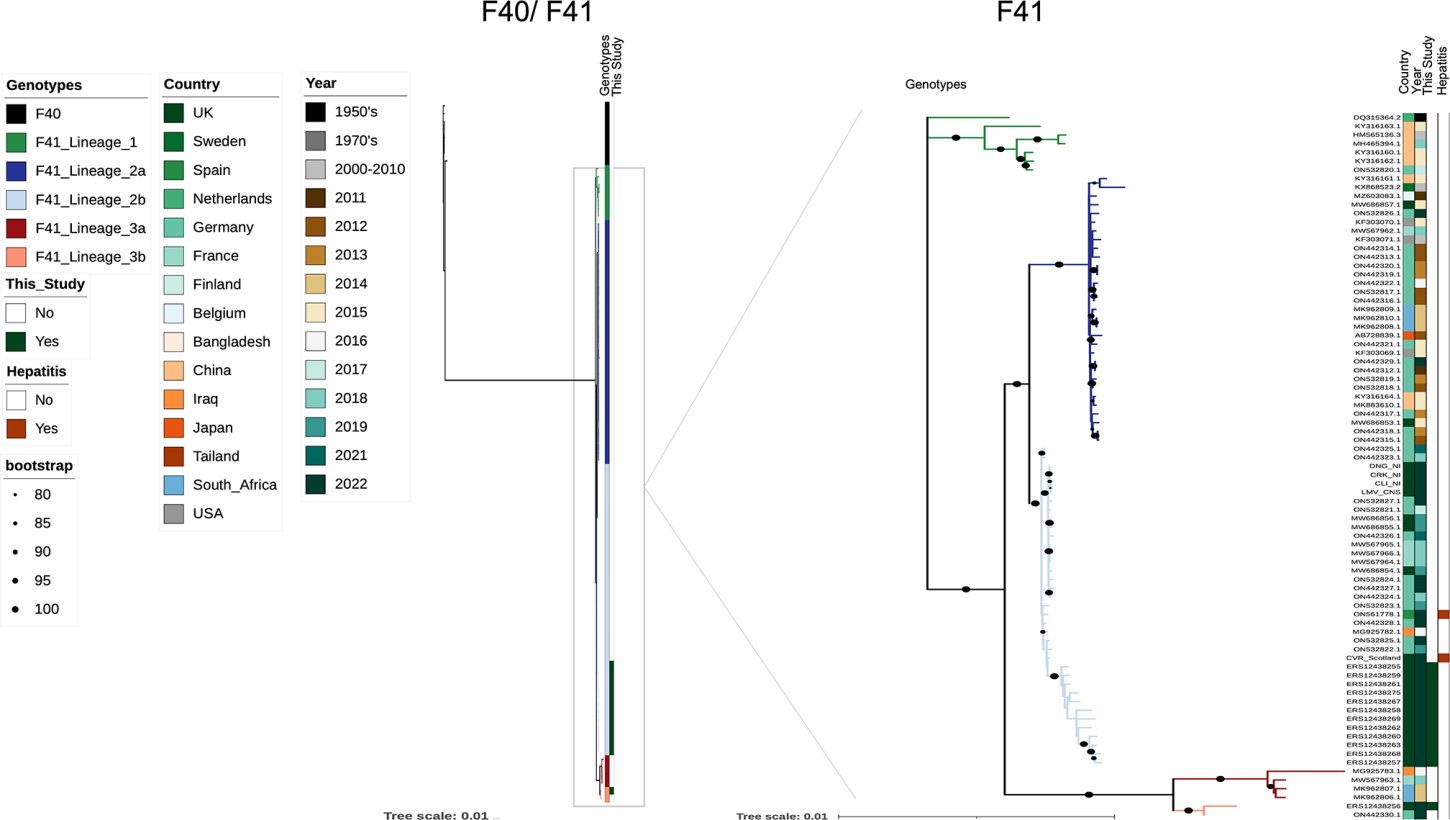
Maximum-likelihood phylogeny of HAdV-F genomes greater than 20 000 bases in length from GenBank (on 27/06/2022). The left tree shows both F40 and F41 genomes, the right tree is zoomed in on F41 genomes for better resolution. Branches with bootstrap support between 80 and 100 % (based on 1000 bootstraps) are marked with a black dot. Sequences generated as part of this study with more than 75 % genome coverage are highlighted on track 3; sequences derived from AHUAC are highlighted on track 4. Lineage structure is adapted from published work [25]. The tree scale indicates nucleotide substitutions/site. Phylogenies are also available to view on Microreact [63]: https://microreact.org/project/bYhycuN63ZToY7348qJHbr-adenoviruscambridge.

Thirteen samples with greater than 75 % genome coverage were used for phylogenetic analysis. Following alignment to all HAdV-F41 genomes available in GenBank on 27/06/22 over 20 kb in length, phylogenetic analysis recapitulated the three-lineage structure of HAdV-F41 diversity (Fig. 4).

There are currently two HAdV-F41 genomes available that are associated with cases of AHUAC, from patients in Spain and Scotland (UK). Both hepatitis-associated sequences form part of widespread European lineage 2b. This lineage has been in circulation since at least 2016 and has been detected both pre- and post-COVID-19 restrictions in Iraq, Germany, Spain, the UK and France. Twelve of the samples sequenced from the East of England region also fell within lineage 2b. One sample (ERS12438275) came from a patient who had a recent history of travel to Dubai; however, it is unclear whether this infection was contracted internationally or locally. Sample ERS12438256 is a member of lineage 3b, first identified in Hannover, Germany, in 2022 [25]. This suggests that at least two lineages (2b and 3b) of HAdV-F41 were present in the UK during January–May 2022.

## Discussion

Here, we show that PrimalScheme overlapping PCR-amplicon-based sequencing [27] of HAdV genomes on the Oxford Nanopore platform is suitable for low *C*_t_/high-virus-load stool samples. Overlapping PCR-amplicon-based sequencing has been successfully deployed for outbreaks of RNA viruses such as Zika and dengue (flaviviruses), Ebola (filovirus) and SARS-CoV-2 (coronavirus) [26, 27, 40, 41]. We applied this method to sequencing a DNA virus, enteric HAdV-F41, during a period of increased case ascertainment and identification of a new virus-associated syndrome (AHUAC) in the UK [13]. Most commercial and widely used PCR primers do not distinguish between HAdV-F40 and -F41; therefore, an amplicon design that has the potential to amplify both types is desirable. Previous studies have used cell culture [42], bait-based enrichment [33] or metagenomic sequencing on the Illumina MiSeq [43] and HiSeq [44] platforms to recover whole HAdV genomes from clinical samples. Evaluating new methods for HAdV sequencing that utilize alternative approaches and sequencing platforms is important for recovering genomic sequences from HAdV-positive clinical samples, including AHUAC cases with a positive HAdV diagnostic result. Sequencing also provides background genomic epidemiological data for ongoing public-health investigations into the relationship between HAdV genetic diversity and disease outcomes following infection. The ability to sequence highly multiplexed viral genomes has the potential to drive down costs and increase sequencing throughput for endemic viruses and during outbreaks [45], as has been shown for SARS-CoV-2 [41, 46], HCV (hepatitis C virus) [47, 48] and Zika virus [27]. Sequencing methods that do not generate large amounts of off-target host genomic data have particular value in public-health and outbreak settings, minimizing the risk that human genomic data may be used to identify a host or link host and sample. These methods also remove the need to clean human reads from sequencing data that are to be publicly shared [49]. Further primer design iterations are ongoing and are expected to reduce amplicon drop out and increase coverage of the hexon region, following the approach used for SARS-CoV-2 [41, 50]. Amplicon-based HAdV sequencing has the potential to be a cost-effective, flexible and high-throughput tool for sequencing species F viruses, while metagenomic [25] and bait-based approaches [23] have advantages of depth and breadth of coverage, sensitivity to low input viral copy numbers and flexibility to detect novel or divergent genotypes [51].

In this study, we sequenced 21 HAdV-positive stool samples from paediatric cases. *C*_t_ values were low in these samples, with a *C*_t_ range of 4.6–15.2, while the *C*_t_ values reported from AHUAC cases were all ≥25 [21]. Metagenomic and target-enriched sequencing of DNA and RNA viruses typically shows a correlation between input virus load or *C*_t_ value and sequence coverage (including RNA and DNA viruses of a range of genome lengths and sequencing methods) [23, 52, 53]. No reads mapped to HAdV-F from the negative control. Given the weak relationship between sample *C*_t_ and sequence depth, and the lack of statistically significant relationship between *C*_t_ and coverage (Fig. 3), the protocol used in this study may need further optimization to increase sequencing success from high-*C*_t_ clinical samples or samples extracted from blood rather than stool. Reduced coverage within the L3 hexon and L4 hexon assembly protein regions suggests future primer design iterations could improve coverage, which was successfully undertaken for SARS-CoV-2 amplicon-based sequencing [41]. We note that one adult blood sample and the NIBSC control material, which was also extracted from a spiked blood sample, did not produce any genomic sequence and had *C*_t_ in the range 23–25. Increasing PCR amplification cycles from 25 to 35 did not generate a product in the blood sample from a viraemic adult, while qPCR-HAdV-negative blood samples did not generate amplicons. Therefore, insufficient amplification or mispriming against host DNA are unlikely to be the cause of the failure to generate HAdV amplicons from blood-derived extracts. This may instead be due to PCR inhibitors present at higher concentrations in blood than in stool, such as antivirals, heparin, EDTA, iron or immunoglobin [54, 55]. Future optimization could include extraction from plasma rather than whole blood. Enzymes intended for blood direct PCR may be beneficial as they are more tolerant of inhibition. Lack of amplification from blood samples may also be due to increased fragmentation of DNA extracted from blood, previously shown to be a factor for adenovirus [56, 57] and other DNA viruses such as human cytomegalovirus [58] in cell-free DNA from human plasma.

In our study of samples received into our laboratory in the East of England from a number of hospital and primary care settings, it is interesting to note that HAdV-F41 was found in multiple paediatric stool samples during the period (January–May 2022), but only rarely detected in adults. This may be because adults are less likely to develop symptomatic disease following infection, which leads to reduced healthcare-seeking behaviour; or because patterns of HAdV-F41 infection are closely linked to age, because of human behaviour or immunological factors. This study cannot rule out that HAdV-F41 infection of adults is widespread but sub-clinical. However, data from the UK in 2014 showed that in older adults, adenovirus was detected in only a small proportion of cases of community onset AGE and these cases were attributed to species C HAdV [59].

This study provides data on the genomic diversity of circulating HAdV-F41 in the UK during a period of increased case ascertainment [12]. Genome sequencing identified that at least two HAdV-F41 lineages were present in East Anglia during the study period. One patient had a history of international travel, but it is unclear whether the lineage 2b infection was contracted in Dubai or the UK. We also identified a second sequence of lineage 3b, first sequenced from a paediatric AGE case in Hannover, Germany, in 2022 [25]. Two genomes of HAdV associated with AHUAC are currently available (shown in dark red in Fig. 4) and both fall within the diversity of HAdV-F41 lineage 2b, which circulated both pre- and post- 2020–2021 COVID-19 restrictions in the UK and Germany. Lineage replacement alone is, therefore, unlikely to account for the apparent increase in AHUAC cases in the UK and elsewhere in early 2022; sequencing of partial hexon genes from AHUAC cases in Alabama, USA, also found multiple F41 lineages to be associated with AHUAC [60]. As cases of AHUAC are in decline across Europe, it is important to capture the diversity of contemporaneously circulating pathogens associated with this condition for future studies [19, 61, 62].

We compared the genome similarity of the newly sequenced genomes from East Anglia, UK, with representatives of closely related lineages (Fig. S2). As well as evidence of decreased similarity in the partial hexon sequence from lineage 3b sample ERS12438275 (also seen in the hexon sequence of lineage 3b F41 from Germany), there is decreased similarity upstream and downstream of the short and long fibre genes. While the majority of variation between adenovirus species and types is concentrated in the hexon, penton and fibre genes, the E3 gene (upstream of the short and long fibre genes) is also highly diverse [55]. Adenovirus species A and F do not produce the E3-19K protein found in other HAdVs [56], but make other unique E3 products (19.4K and 31.6K) with immune modulatory functions. The species F E3 region appears to have a role in preventing infected cells from signalling to natural killer (NK) cells via intracellular sequestration of MICB [57]. Lineage 3b viruses show evidence of decreased genome similarity to other F41 lineages in this region. We also note increased diversity in the E4 ORF6/7 region, in line with other groups [25], which plays a partially redundant role in viral growth and late viral protein synthesis [58]. Further analysis of F41 genomes sequences from a wider representation of countries will allow researchers to identify whether variation within regions such as E3 is important for immune modulation, given the variability in human *MICB* alleles worldwide [59].

In conclusion, overlapping PCR-amplicon sequencing on the Nanopore MinION platform is an appropriate method for generating HAdV-F41 sequence data from stool samples, particularly where *C*_t_ values are low. HAdV genome sequencing allows us to identify virus lineages circulating before and after the 2020–2021 phase of the COVID-19 pandemic in the UK, which will inform future studies of HAdV-F41 diversity and disease association. Understanding the role of adenovirus and other DNA viruses in novel disease presentations such as non-A–E paediatric hepatitis will require a global effort to characterize the genomics of common childhood infections such as HAdV-F41, and multiple sequencing strategies will be required to enable this.

## Supplementary Data

Supplementary material 1

Supplementary material 2
