# Supplementary material for: Multiplex MinION sequencing suggests enteric adenovirus F41 genetic diversity comparable to pre-COVID-19 era
Source: Microb Genom. 2023 Jan 6;9(1):mgen000920. doi: 10.1099/mgen.0.000920 (PMC9973849; doi:10.1099/mgen.0.000920)
Supplement: Supplementary material 1 [file mgen-9-920-s001.pdf]

MGEN-D-22-00233 Supplementary methods and figure

Short title: Screening and sequencing adenovirus F41

Title: Enteric adenovirus F41 genetic diversity comparable to pre-COVID-19 era: validation of a multiplex amplicon-MinION sequencing method

Authors

Mailis Maes, Fahad Khokhar, Sam AJ Wilkinson, Andrew D Smith, Ganna Kovalenko, Gordon Dougan, Joshua Quick;, Nicholas J Loman;, Stephen Baker, Martin D Curran, Jordan P Skittrall, Charlotte J Houldcroft.

Primer design - overlapping amplicons

Whole genome sequences for 125 HAdV-F isolates over 20kb in length were downloaded from Genbank. Of these 113 genomes (112 F41 genomes and the F reference genome NC_001454) were used for primer design (supp table 1). A masked reference (variant positions masked with IUPAC ambiguity codes) was generated using (https://github.com/ChrisgKent/NMaskGen_Snakemake). Using this mask, a scheme consisting of 92 1.2 kb overlapping amplicons were designed using a version of the PrimalScheme software modified to output 4 pools (https://github.com/quick-lab/HAdV/blob/main/HAdV-F41/v1.0/HAdV-F41_1200jh.primer.bed) (supp table 2). This so called 'Jackhammer' approach was added to provide additional redundancy against amplification failures.

PCR amplification of viral genomes

Primer stocks were resuspended to 100µM in 1xTE then pooled in an equimolar fashion into 4 primer pools. Primer pools were then diluted in molecular grade water to generate 10 µM working stocks. HAdV-F positive extracts were amplified using PCR in four pools (supp table 3). Reactions were performed in 25 µl volumes using Q5 Hot Start High-Fidelity 2X Master Mix (NEB), 2.5 µl of the extracted DNA with four primer pools in separate tubes. The reaction mix was incubated with the following conditions: 98°C for 30 sec as a heat activation cycle then 25 cycles of 98°C for 15 sec and 65°C for 5 min. 25 cycles of amplification were performed as 21/24 samples had Ct values <16 [3].

The four PCR reactions were then pooled for each sample as follows: 2.5 µl of each pool was added to 90 µl of nuclease-free water, for a final volume of 100 µl. 3.3 µl of this pool was then used for the library preparation.

PCR product size of pooled PCR reactions were visualised on the 4200 TapeStation System using the genomic DNA reagents and genomic DNA ScreenTape. Both samples extracted from blood did not give the right size band. In order to exclude mispriming to host DNA or lack of amplification due to low viral load in these samples we ran an additional PCR with Adenovirus negative bloods one of positive blood samples and a positive control stool sample for 25 and 35 cycles. (suppl. Fig 1)

Library preparation

Library preparation followed the SARS-CoV-2 LoCost clean-up and barcoding methods [3, 4], using the SQK-LSK109 ligation sequencing kit with EXP-NBD104 and EXP-NBD114 native barcodes from Oxford Nanopore Technologies (Oxford, UK).

End preparation was performed on an individual sample basis in a 10µl volume as follows: 3.3µl of pooled PCR products, 5µl nuclease free water, 1.2µl Ultra II End Prep Reaction

Buffer (NEB) and 0.5µl Ultra II End Prep Enzyme Mix (NEB). Following a room temperature (22 °C) incubation for 15 minutes, samples were incubated at 65 °C for 15 minutes and placed on ice for 1 minute. Barcoding was performed on an individual sample basis in a 10µl volume as follows: 0.75µl end-prepared sample from the previous step, 1.25µl NBXX barcode (ONT), 5µl Blunt/TA Ligase Master Mix (NEB), 3µl nuclease free water. Following a room temperature (22 °C) incubation for 20 minutes, samples were incubated at 65 °C for 10 minutes and placed on ice for 1 minute. All samples were then pooled using 10µl from each native barcoding reaction. The library was then cleaned up using a 0.4X volume of AMPure XP SPRI beads (Beckman), with a 5 minute room temperature (22 °C) incubation on the bench and a 2 minute incubation on a magnetic rack. Beads were washed in 250µl SFB (ONT) and the incubation on the bench and magnetic rack was repeated. A second SFB wash and incubation was then repeated as before. Samples were then washed in 200µl of room-temperature 70% ethanol, which was then discarded. The library was then resuspended in 30µl 10 mM Tris pH 8.0 and quantified using the Qubit 4 Fluorometer (Invitrogen), following the manufacturer's protocol.

AMII adapter ligation was performed a 50µl volume as follows: 30µl barcoded amplicon pool, 10µl NEBNext Quick Ligation Reaction Buffer (5x) (NEB), 5µl Adapter Mix (AMII) (ONT), 5µl Quick T4 DNA Ligase (NEB). Samples were incubated at room temperature for 20 minutes and cleaned up using a 1:1 volume of SPRI beads. Samples were incubated for 5 minutes at room temperature (22 °C) on the bench and 2 minutes on a magnetic rack. Beads were washed in 250µl SFB (ONT) and the incubation on the bench and magnetic rack was repeated. A second SFB wash and incubation was then repeated as before. Samples were then resuspended in 15µl EB (ONT) and incubated for 2 minutes on a magnetic rack. The final library was then eluted and transferred to a new 1.5ml Eppendorf tube, and quantified as before.

Sequencing

The final pooled library was loaded onto a FLO-MIN106D R9.4.1 flow cell and sequencing was performed on a GridION Mk1 platform for 24 hours using MinKNOW version v22.03.4. With Guppy v6.0.7 integrated into MinKNOW, a live super-accuracy basecalling model with a minimum Q score filtering value of 10 and demultiplexing requiring barcodes on both ends of the reads was applied. The sequencing run generated 18GB of data and a total of 1.22 million reads with an estimated N50 length of 990.

Genome assembly

A modified version (https://github.com/BioWilko/fieldbioinformatics/tree/19c0e5c459d8f9730999eb3623ba5e7b702dc2e9) of the ARTIC fieldbioinformatics pipeline (https://github.com/artic-network/fieldbioinformatics) was utilised to process and assemble the raw read data, on the MRC-CLIMB-BIG-DATA platform [5]. To allow the pipeline to assemble genomes for both F40 and 41 types, a pseudoreference approach was used. This pseudoreference was generated by: aligning all reads to the F reference genome NC_001454 (minimap2 2.2.4) [6], performing a naive pileup, variant calling (ignoring indels to maintain reference coordinates), and consensus generation (all with bcftools 1.13) [7]. After generating the pseudoreference the fieldbioinformatics medaka workflow proceeded as previously described [8] calling all variants against the pseudoreference instead of the HAdV-F RefSeq NC_001454. The minimum depth for base calling was 20x.

Genome coverage of trimmed aligned reads (primertrimmed.rg.sorted.bam) was calculated using Bedtools [9] v2.30.0 genomecov with the -d option [9]. Coverage and depth plots were made using Matlab R2021a (MathWorks, Cambridge, UK) (supp file 1).

References

1. **Clark TW, Medina MJ, Batham S, Curran MD, Parmar S, *et al.*** Adults hospitalised with acute respiratory illness rarely have detectable bacteria in the absence of COPD or pneumonia; viral infection predominates in a large prospective UK sample. *J Infect* 2014;69:507–515.
2. **Tiemessen CT, Nel MJ**. Detection and typing of subgroup F adenoviruses using the polymerase chain reaction. *J Virol Methods* 1996;59:73–82.
3. nCoV-2019 sequencing protocol v3 (LoCost). https://www.protocols.io/view/ncov-2019-sequencing-protocol-v3-locost-bp2l6n26rgqe/v3 (accessed 27 June 2022).
4. **Tyson JR, James P, Stoddart D, Sparks N, Wickenhagen A, *et al.*** Improvements to the ARTIC multiplex PCR method for SARS-CoV-2 genome sequencing using nanopore. *bioRxiv Prepr Serv Biol* 2020;2020.09.04.283077.
5. **Connor TR, Loman NJ, Thompson S, Smith A, Southgate J, *et al.*** CLIMB (the Cloud Infrastructure for Microbial Bioinformatics): an online resource for the medical microbiology community. *Microb Genomics* 2016;2:e000086.
6. **Li H**. Minimap2: pairwise alignment for nucleotide sequences. *Bioinformatics* 2018;34:3094–3100.
7. **Danecek P, Bonfield JK, Liddle J, Marshall J, Ohan V, *et al.*** Twelve years of SAMtools and BCFtools. *Gigascience*;10. Epub ahead of print 16 February 2021. DOI: 10.1093/gigascience/giab008.
8. **Loman N, Rowe W, Rambaut A**. nCoV-2019 novel coronavirus bioinformatics protocol. https://artic.network/ncov-2019/ncov2019-bioinformatics-sop.html (2020, accessed 6 May 2020).
9. **Quinlan AR, Hall IM**. BEDTools: a flexible suite of utilities for comparing genomic features. *Bioinformatics* 2010;26:841–842.
10. **Tahmasebi R, Luchs A, Tardy K, Hefford PM, Tinker RJ, *et al.*** Viral gastroenteritis in Tocantins, Brazil: characterizing the diversity of human adenovirus F through next-generation sequencing and bioinformatics. *J Gen Virol* 2020;jgv001500.
11. **Götting J, Cordes AK, Steinbrück L, Heim A**. Molecular Phylogeny of human adenovirus type 41 lineages. *bioRxiv* 2022;2022.05.30.493978.
12. **UKHSA**. *Investigation into acute hepatitis of unknown aetiology in children in England Technical briefing 2*. 2022.
13. **Katoh K, Rozewicki J, Yamada KD**. MAFFT online service: multiple sequence alignment, interactive sequence choice and visualization. *Brief Bioinform*. Epub ahead of print 6 September 2017. DOI: 10.1093/bib/bbx108.
14. **Tamura K, Stecher G, Kumar S**. MEGA11: Molecular Evolutionary Genetics Analysis Version 11. *Mol Biol Evol* 2021;38:3022–3027.
15. **Hoang DT, Chernomor O, Von Haeseler A, Minh BQ, Vinh LS**. UFBoot2: Improving the Ultrafast Bootstrap Approximation. *Mol Biol Evol* 2018;35:518–522.
16. **Nguyen LT, Schmidt HA, Von Haeseler A, Minh BQ**. IQ-TREE: A fast and effective stochastic algorithm for estimating maximum-likelihood phylogenies. *Mol Biol Evol* 2015;32:268–274.
17. **Letunic I, Bork P**. Interactive Tree Of Life (iTOL) v5: an online tool for phylogenetic tree display and annotation. *Nucleic Acids Res* 2021;49:W293–W296.
18. **Samson S, Lord É, Makarenkov V**. SimPlot++: a Python application for representing sequence similarity and detecting recombination. *Bioinformatics* 2022;38:3118–3120.

Supplementary figure 1

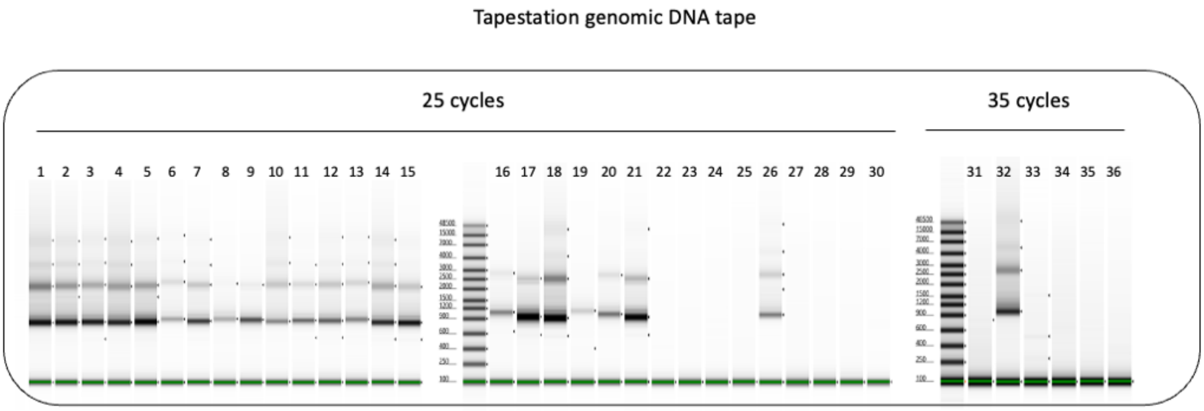

Lane	Sample	Lane	Sample	Lane	Sample	Lane	Sample	Lane	Sample	Lane	Sample
1	NB01(Stool)	7	NB07(Stool)	13	NB13(Stool)	19	NB19(Stool)	25	NB22 repeat PCR (positive Blood)	31	NB22 repeat PCR (positive Blood)
2	NB02(Stool)	8	NB08(Stool)	14	NB14(Stool)	20	NB20(Stool)	26	NB04 repeat PCR(Positive Stool)	32	NB04 repeat PCR(Positive Stool)
3	NB03(Stool)	9	NB09(Stool)	15	NB15(Stool)	21	NB21(Stool)	27	Negative (Blood)	33	Negative (Blood)
4	NB04(Stool)	10	NB10(Stool)	16	NB16(Stool)	22	NB22(Blood)	28	Negative (Blood)	34	Negative (Blood)
5	NB05(Stool)	11	NB11(Stool)	17	NB17(Stool)	23	NB23(NIBSC from blood)	29	Negative (Blood)	35	Negative (Blood)
6	NB06(Stool)	12	NB12(Stool)	18	NB18(Stool)	24	NB24(H20)	30	H20	36	H20

Supp fig 1: Plot showing product sizes for each sample after pooling PCR amplicons from pools V1-V4. On the left of the plot are products after 25 cycles of amplification. On the right are selected samples after 35 samples of amplification. Plot made on a Tapestation using the Genomic DNA ScreenTape (catalogue number 5067- 5365) with Genomic DNA Reagents (Ladder and Sample Buffer) (catalogue number 5067- 5366).

Supplementary figure 2

A

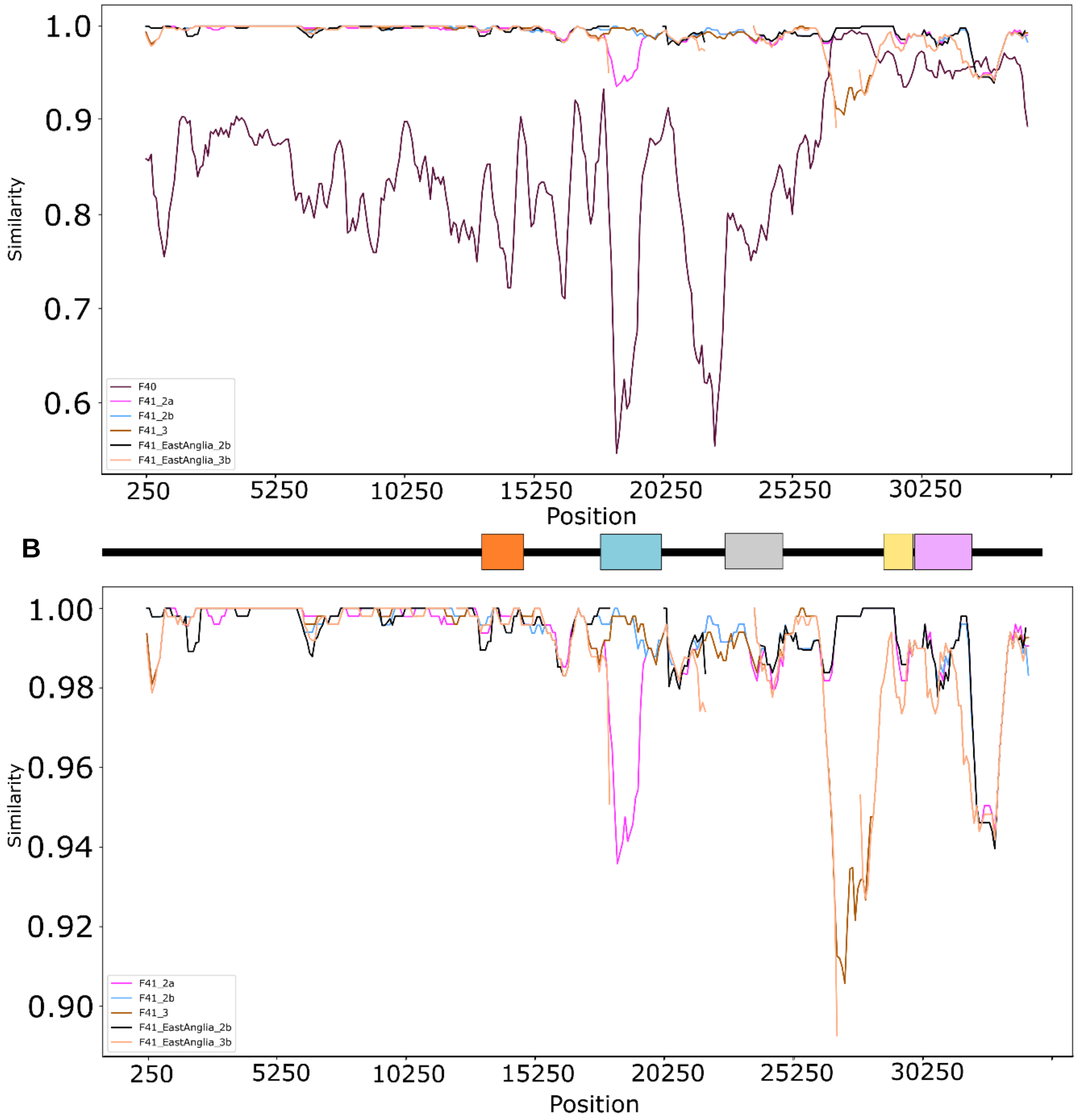

Supp fig 2: **A.** Similarity plot showing nucleotide similarity between the query sequence, F41 lineage 1 (DQ315363.2), and four other HAdV-Fs: F40 (NC001454.1) in dark red; lineage 2a (KX868523.2) in pink; lineage 2b (MG925782) in bright blue; lineage 3b (ON442330) in brown; the 12 East Anglia lineage 2b genomes (black) and the East Anglian 3b genome (orange). Genome position is shown on the X axis and similarity is shown on the Y axis. The window size was 500bp and the step size was 100bp. The four capsid proteins are shown below the X axis: penton (orange), hexon (blue), short fiber (yellow) and long fiber (purple);

the hexon assembly protein is shown in grey. Reduced similarity between F40 and F41 genomes is seen in parts of the L3 hexon (blue) and L4 hexon assembly (grey) regions of the genome. **B**. Similarity plot showing nucleotide similarity between the query sequence, F41 lineage 1 (DQ315363.2), and four other HAdV-Fs: F40 (NC001454.1) in dark red; lineage 2a (KX868523.2) in pink; lineage 2b (MG925782) in bright blue; lineage 3b (ON442330) in brown; the 12 East Anglia lineage 2b genomes (black) and the East Anglian 3b genome (orange). Decreased similarity between lineage 3b and lineage 1 and 2 F41 viruses can be seen upstream (gene E4) and downstream of the short and long fibers.

Supplementary table 1: Accessions of adenovirus genomes used for primer design

MW686857.1
MW686856.1
MW686855.1
MW686854.1
MW686853.1
MW567966.1
MW567965.1
MW567964.1
MW567963.1
MW567962.1
MT791001.1
MT790999.1
MT790998.1
MT797134.1
MT797133.1
MT797132.1
MT797131.1
MT797130.1
MT797129.1
MT797128.1
MT797127.1
MT797126.1
MT797125.1
MT797124.1
MT797123.1
MT797122.1
MT797121.1
MT797120.1
MT797119.1
MT797118.1
MT797117.1
MT797116.1
MT797115.1
MT797114.1
MT797113.1
MT797112.1
MT797111.1
MT797110.1
MT797109.1
MT797108.1
MT797107.1

MT797106.1
MT797105.1
MT797104.1
MT797103.1
MT797102.1
MT797101.1
MT797100.1
MT797099.1
MT826902.1
MT826901.1
MT826900.1
MT826899.1
MT826898.1
MT826897.1
MT826896.1
MT826895.1
MT826894.1
MT826893.1
MT826892.1
MT826891.1
MT826890.1
MT826889.1
MT826888.1
MT826887.1
MT826886.1
MT826884.1
MT826885.1
MT815574.1
MT815573.1
MT815572.1
MT815571.1
MT815570.1
MT815569.1
MT815568.1
MT815567.1
MT815566.1
MT815565.1
MT815564.1
MT815563.1
MT815562.1
MT815561.1
MT815560.1

MT815559.1
MT815558.1
MT815557.1
MT815556.1
MT777457.1
MT777456.1
MT777455.1
MT777454.1
MT777453.1
MK883610.1
MK962810.1
MK962809.1
MK962808.1
MK962807.1
MK962806.1
MH465394.1
KX868523.2
MG925783.1
MG925782.1
KY316164.1
KY316163.1
KY316162.1
KY316161.1
KY316160.1
KF303071.1
KF303070.1
KF303069.1
AB728839.1
HM565136.3
DQ315364.2
NC_001454

Supplementary table 2: PCR primers to generate HAdV-F sequencing amplicons

HAdV-F41_1200jh.primer.bed.xlsx

Supplementary table 3: PCR mix for the four primer pools

**Component**	**Reaction 1**	**Reaction 2**	**Reaction 3**	**Reaction 4**
Q5 Hot Start High-Fidelity 2X Master Mix	12.5 µl	12.5 µl	12.5 µl	12.5 µl
V1 primer pool	4 µl	0 µl	0 µl	0 µl
V2 primer pool	0 µl	4 µl	0 µl	0 µl
V3 primer pool	0 µl	0 µl	4 µl	0 µl
V4 primer pool	0 µl	0 µl	0 µl	4 µl
Nuclease-free water	6 µl	6 µl	6 µl	6 µl
Sample DNA	2.5 µl	2.5 µl	2.5 µl	2.5 µl

Supp table 4: Sample metadata and accessions

Sample	Barcode	Sample type	HAdV Ct	HAdV-F Ct	Coverage	Depth	ERR	ERS
**Stool1**	NB01	Feces	7.34	5.25	77.20	291.1	ERR9939847	ERS12438255
**Stool2**	NB02	Feces	6.29	4.87	81.45	390.1	ERR9939848	ERS12438256
**Stool3**	NB03	Feces	6.39	10.85	79.90	292.6	ERR9939849	ERS12438257
**Stool4**	NB04	Feces	6.54	4.58	85.40	395.1	ERR9939850	ERS12438258
**Stool5**	NB05	Feces	8.78	6.89	79.71	204.0	ERR9939851	ERS12438259
**Stool6**	NB06	Feces	7.16	5.1	79.90	257.8	ERR9939852	ERS12438260
**Stool7**	NB07	Feces	9.94	7.43	78.10	148.8	ERR9939853	ERS12438261
**Stool8**	NB08	Feces	6.88	5.81	76.36	295.2	ERR9939854	ERS12438262
**Stool9**	NB09	Feces	10.66	10.07	88.06	324.4	ERR9939855	ERS12438263
**Stool10**	NB10	Feces	8.79	7.49	73.33	108.4	ERR9939856	ERS12438264
**Stool11**	NB11	Feces	9.93	8.11	68.18	72.6	ERR9939857	ERS12438265
**Stool12**	NB12	Feces	8.8	6.67	71.28	95.7	ERR9939858	ERS12438266
**Stool13**	NB13	Feces	9.59	7.99	75.27	144.6	ERR9939859	ERS12438267
**Stool14**	NB14	Feces	6.28	8.46	79.90	252.5	ERR9939860	ERS12438268
**Stool15**	NB15	Feces	8.36	8.04	78.75	158.0	ERR9939861	ERS12438269
**Stool16**	NB16	Feces	9.94	9.08	48.42	42.7	ERR9939862	ERS12438270
**Stool17**	NB17	Feces	13.41	12.34	71.77	90.6	ERR9939863	ERS12438271
**Stool18**	NB18	Feces	8.13	8.55	47.97	50.7	ERR9939864	ERS12438272
**Stool19**	NB19	Feces	10.2	15.2	57.97	53.0	ERR9939865	ERS12438273
**Stool20**	NB20	Feces	10.57	10.77	64.92	79.5	ERR9939866	ERS12438274
**Stool21**	NB21	Feces	11	10.65	80.49	354.0	ERR9939867	ERS12438275
**Blood Isolate**	NB23	Blood	N/A	24.6	0.00	0.0	ERR9939868	ERS12438276
**NIBSC control**	NB22	Blood	N/A	23.1	0.00	0.0	ERR9939869	ERS12438277
**H2O**	NB24	H2O	N/A	N/A	0.00	0.0	ERR9939870	ERS12438278
								ERA16457764

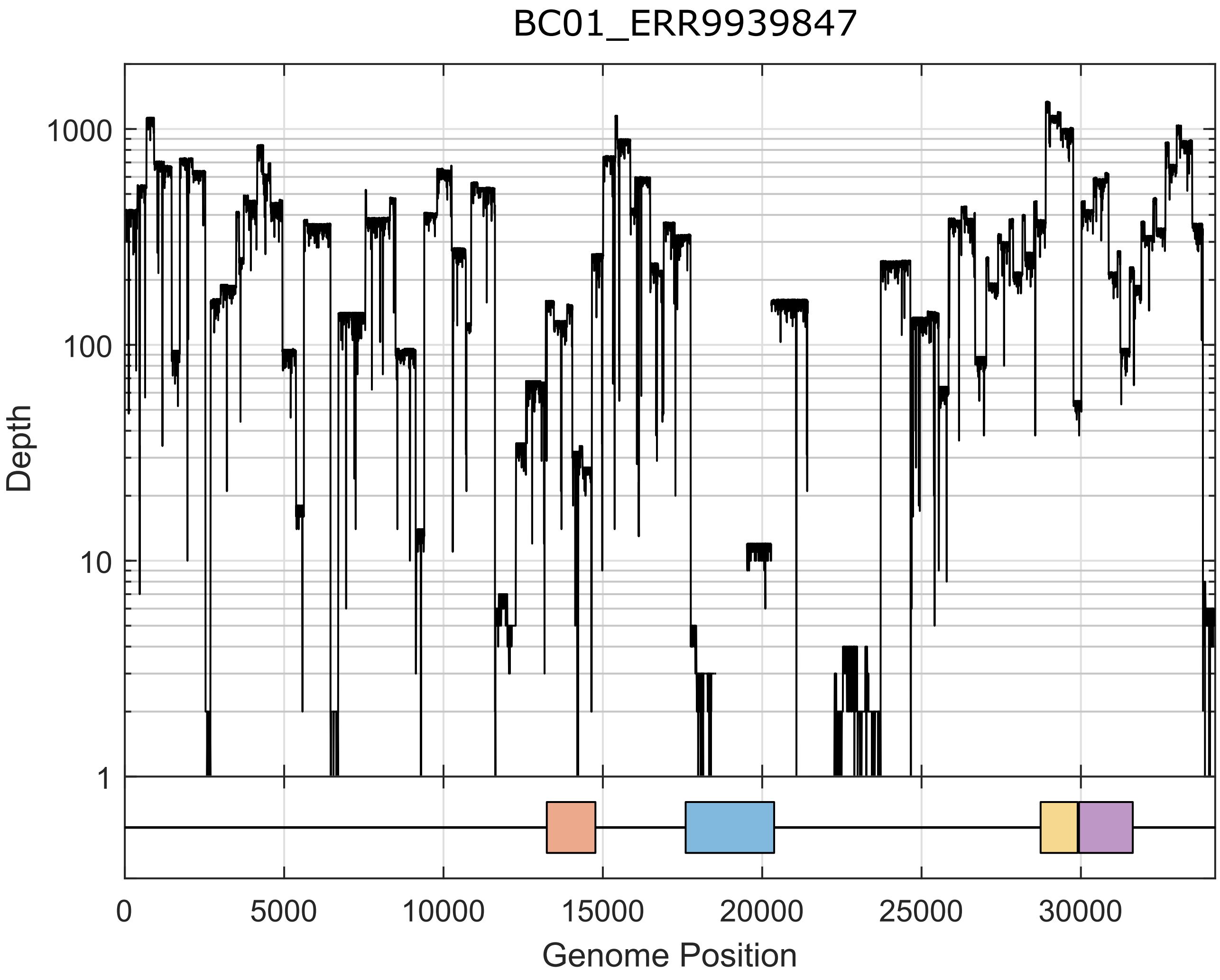
BC01_ERR9939847
Depth
1000
100
10
1
0
5000
10000
15000
20000
25000
30000
Genome Position

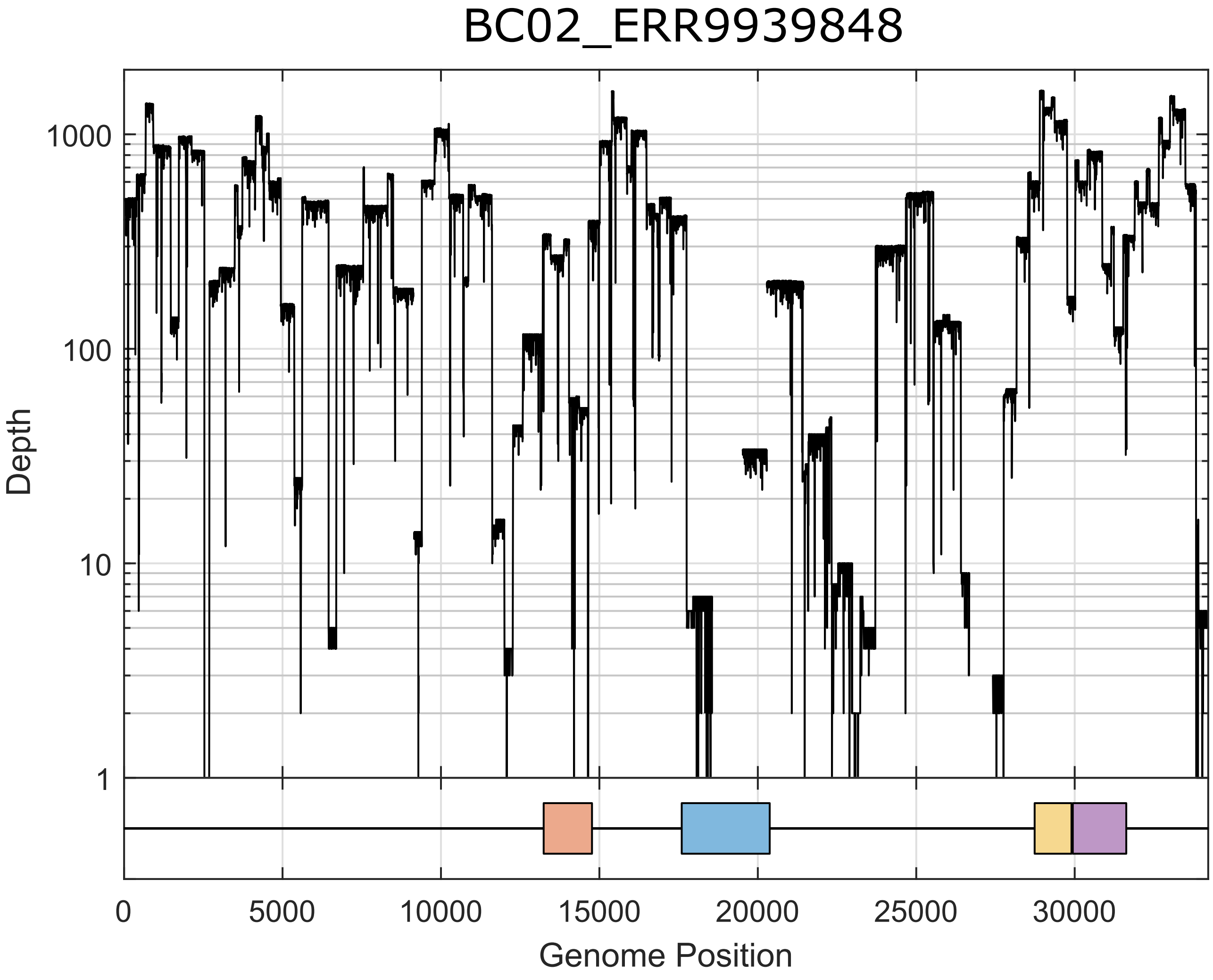

BC02_ERR9939848
1000
100
10
1
Depth
0
5000
10000
15000
20000
25000
30000
Genome Position

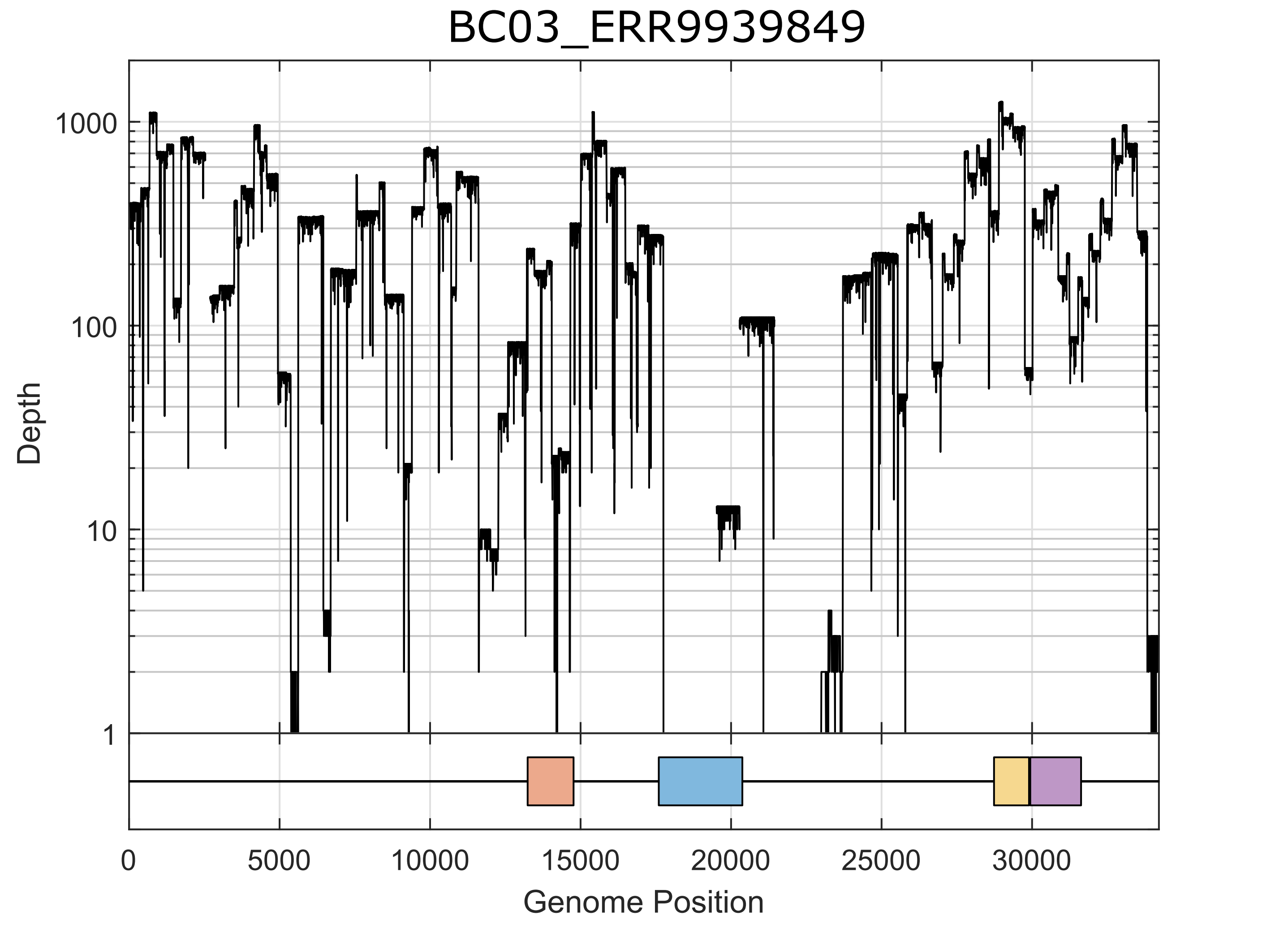
BC03_ERR9939849
1000
100
10
1
Depth
0
5000
10000
15000
20000
25000
30000
Genome Position

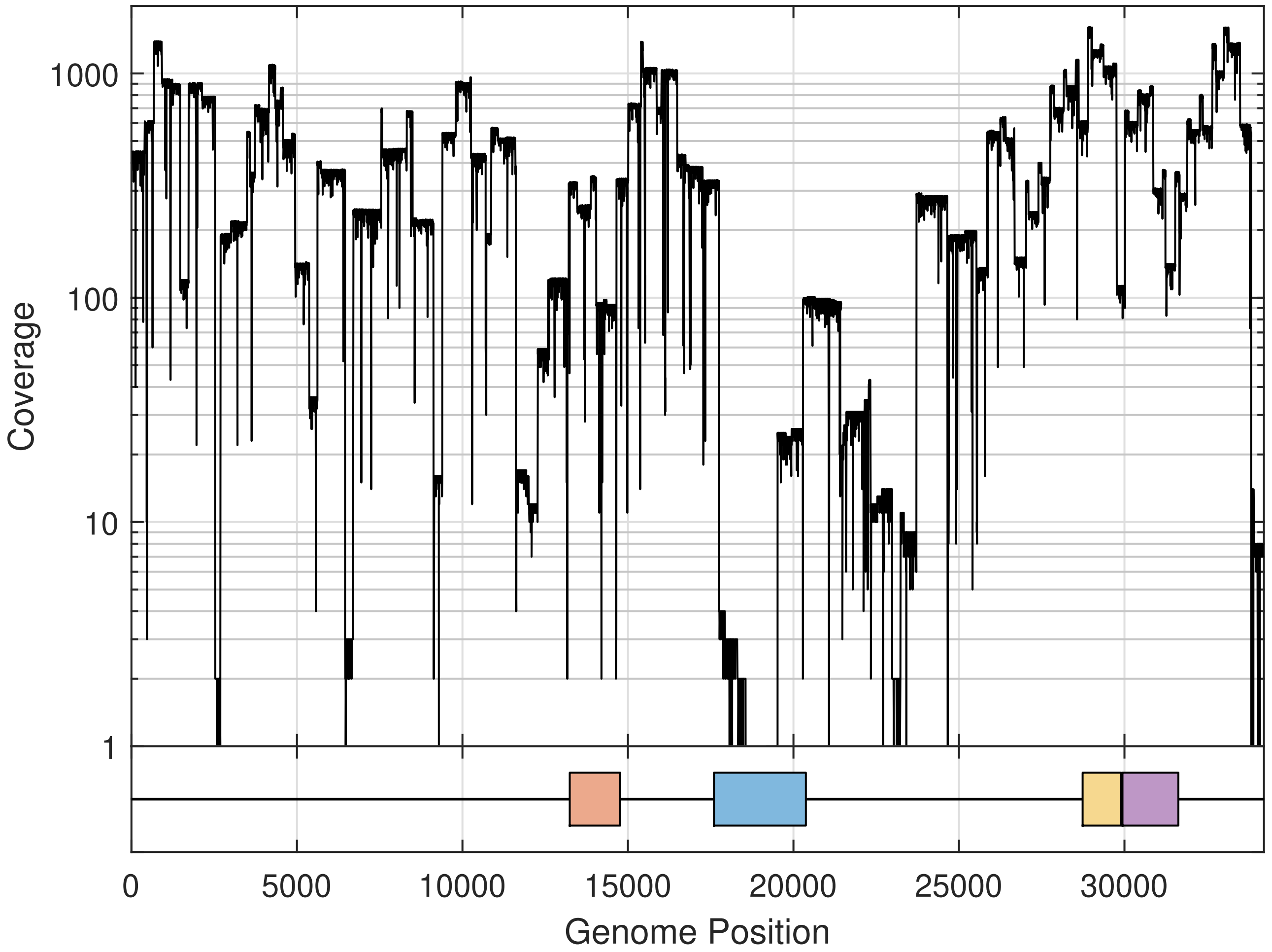

Coverage
1000
100
10
1
0
5000
10000
15000
20000
25000
30000
Genome Position

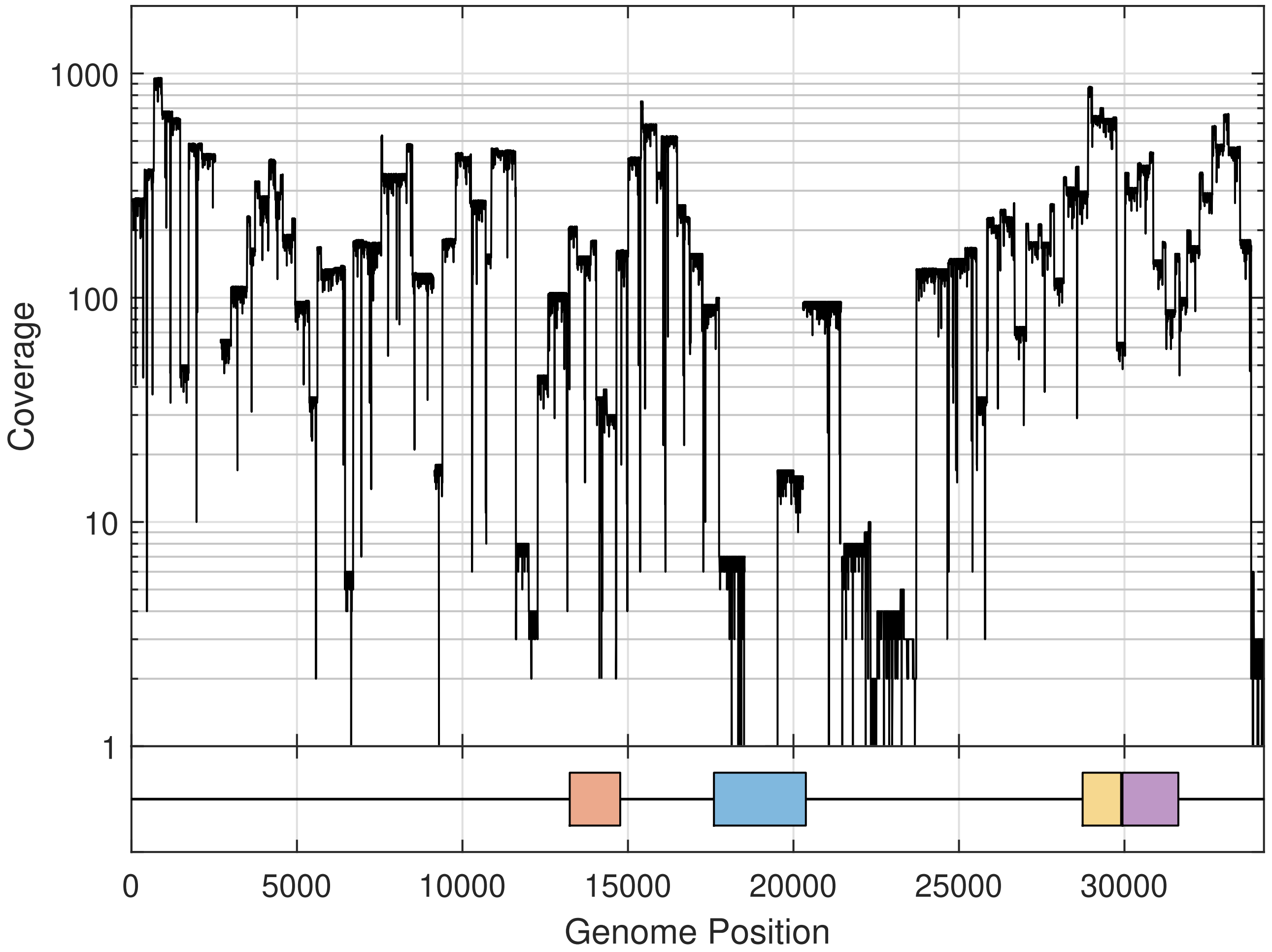

Coverage
1000
100
10
1
0
5000
10000
15000
20000
25000
30000
Genome Position

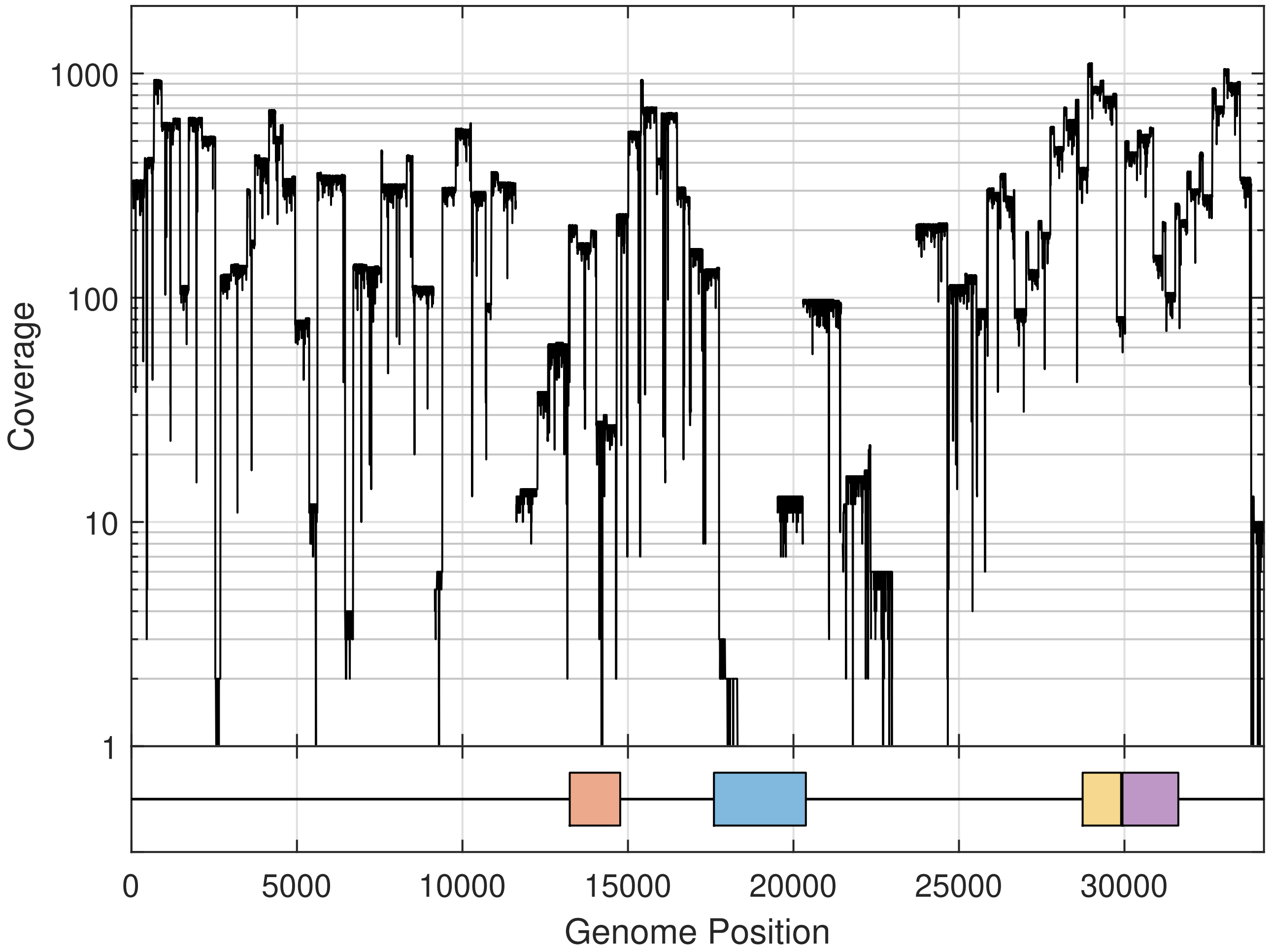

1000
100
10
1
Coverage
0
5000
10000
15000
20000
25000
30000
Genome Position

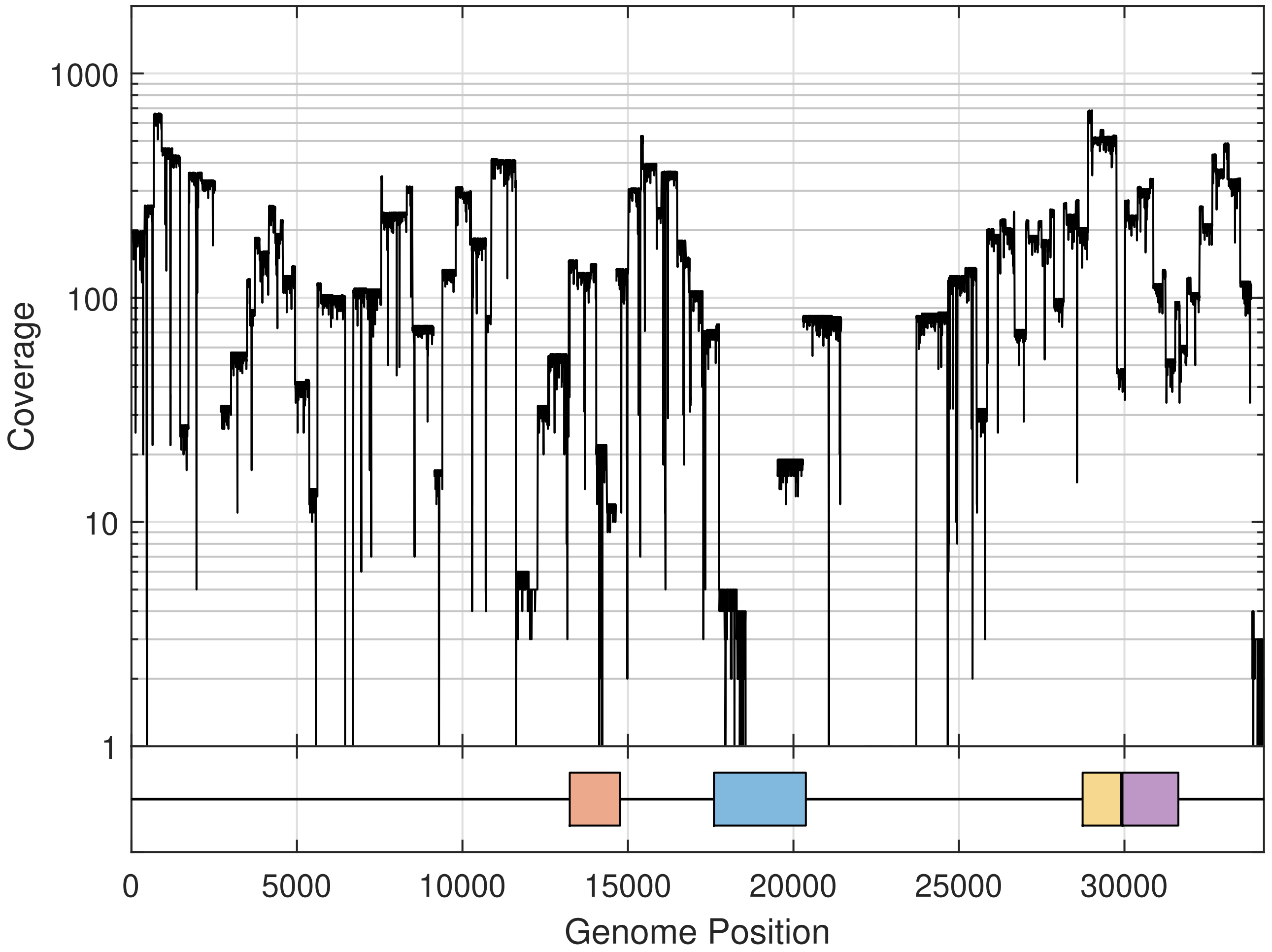

Coverage
1000
100
10
1
0
5000
10000
15000
20000
25000
30000
Genome Position

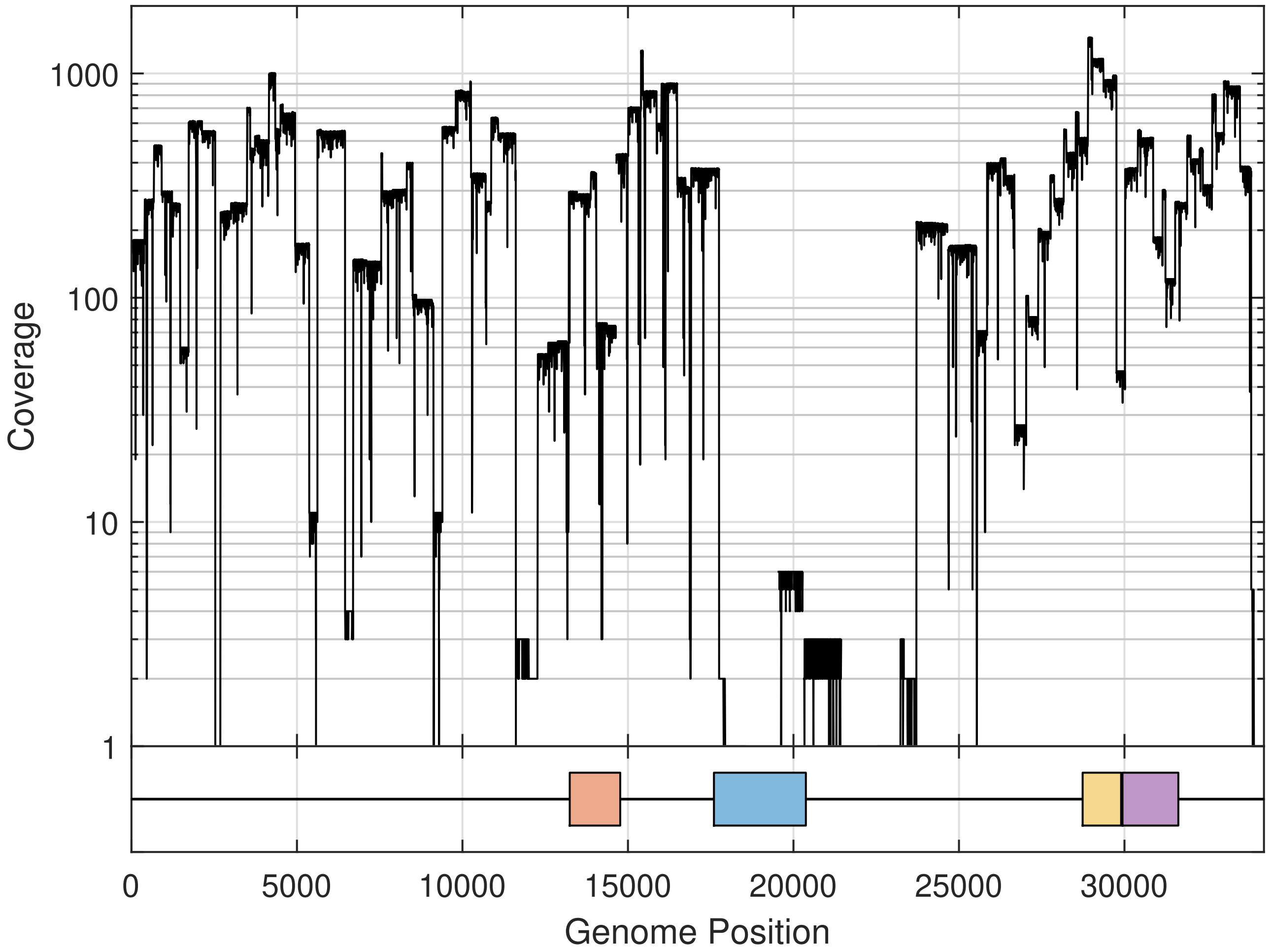

1000
100
10
1
Coverage
0
5000
10000
15000
20000
25000
30000
Genome Position

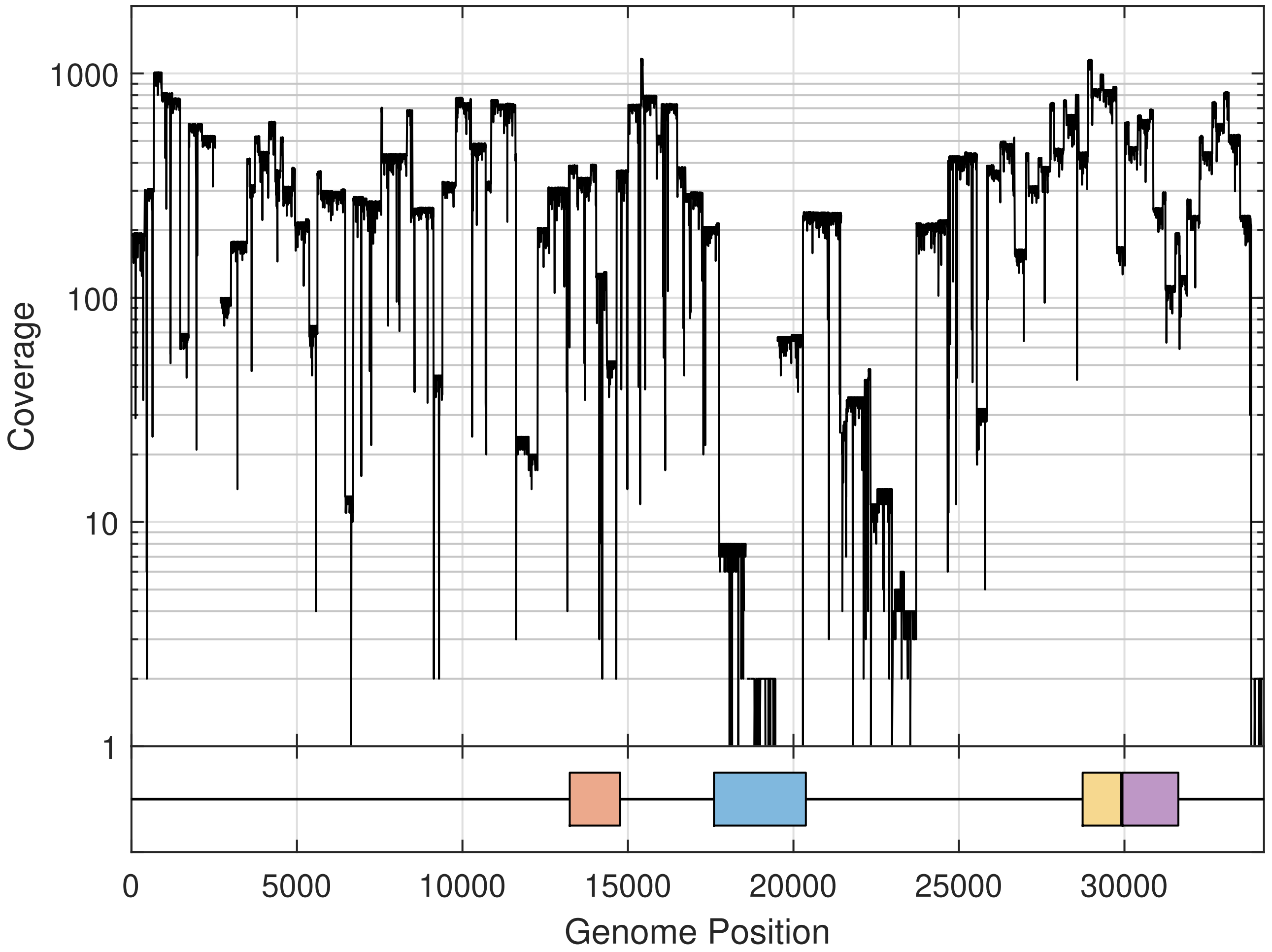

Coverage
1000
100
10
1
0
5000
10000
15000
20000
25000
30000
Genome Position

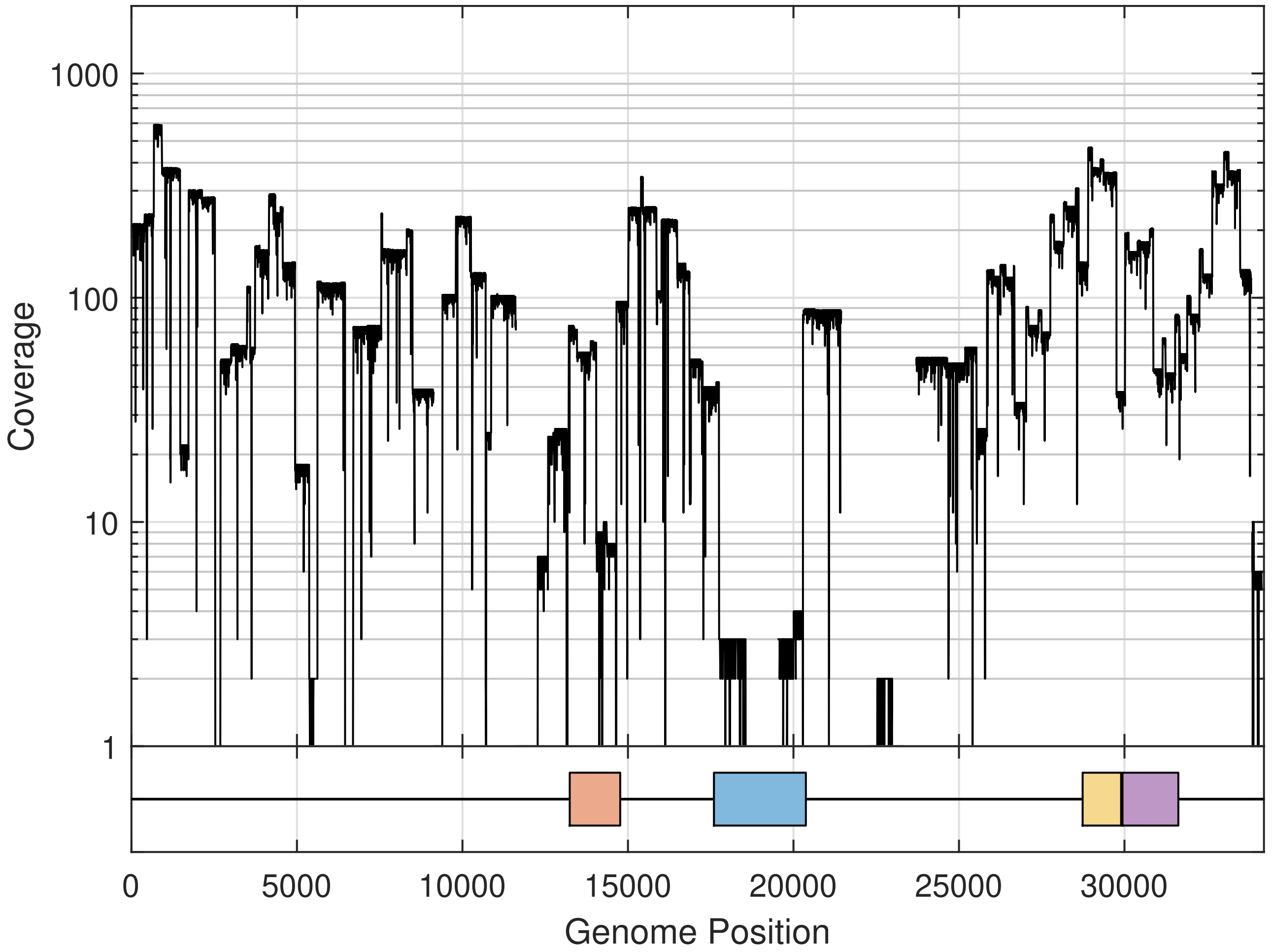

1000
100
10
1
Coverage
0
5000
10000
15000
20000
25000
30000
Genome Position

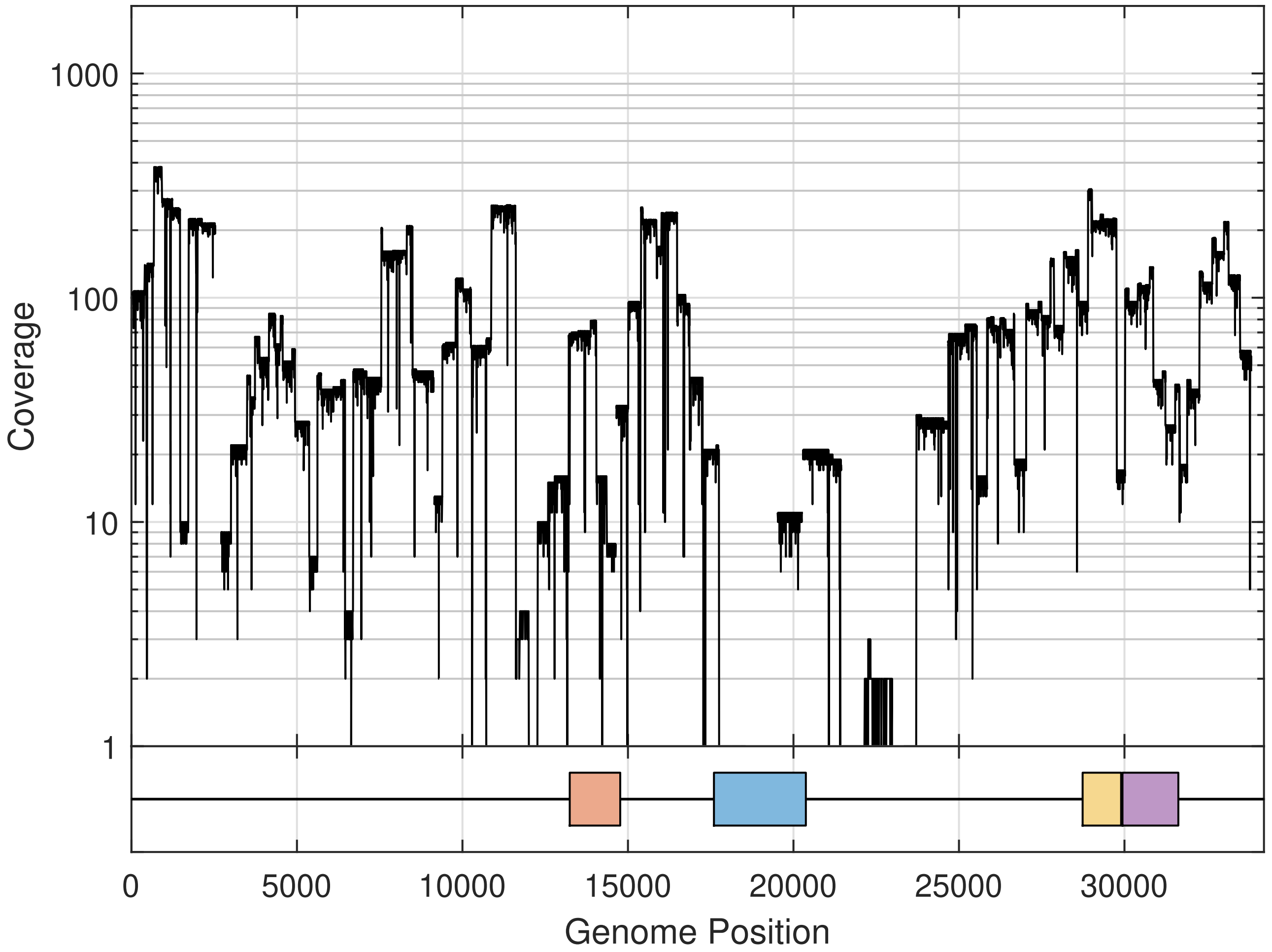

1000
100
10
1
Coverage
0
5000
10000
15000
20000
25000
30000
Genome Position

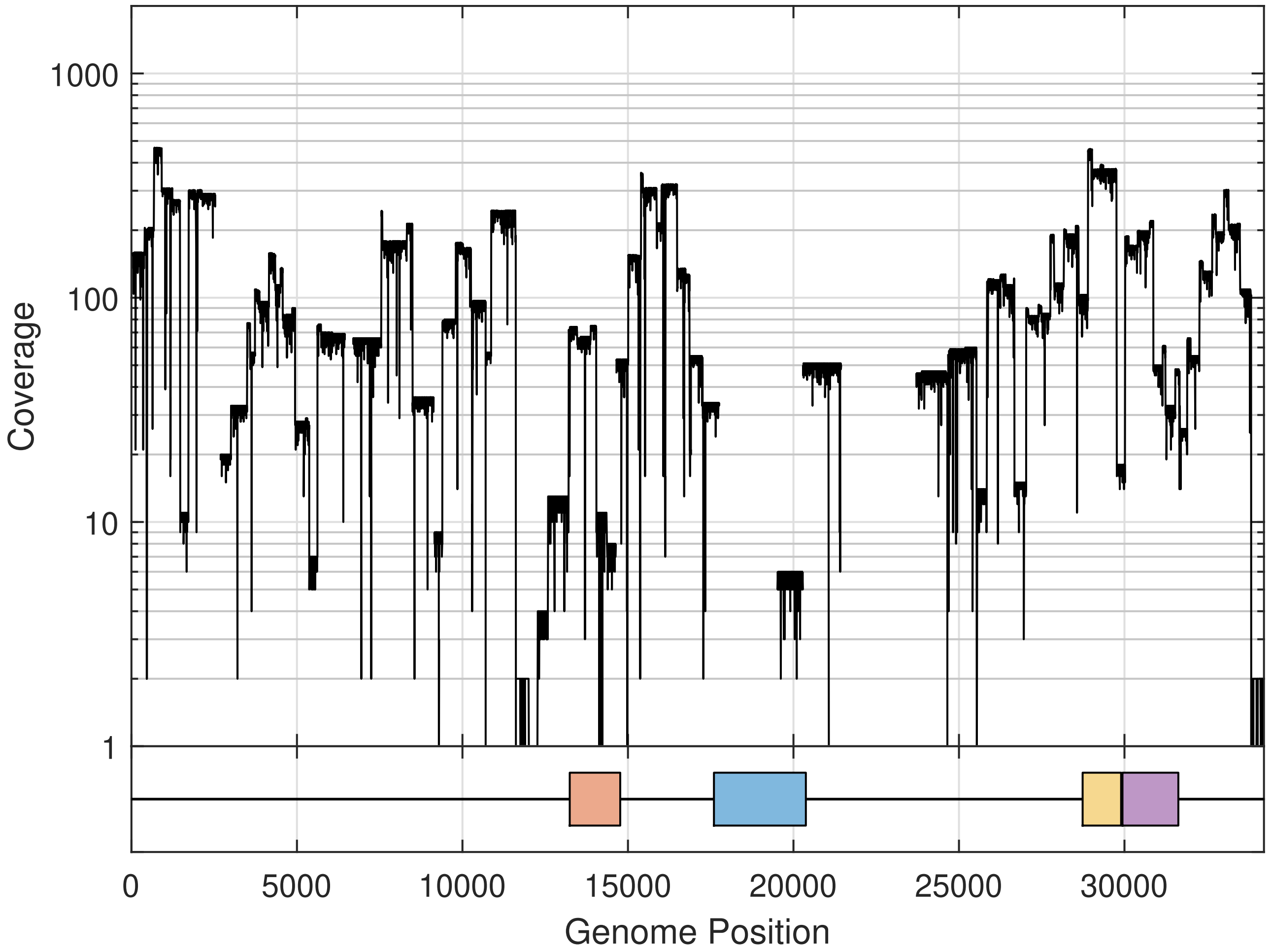

Coverage
1000
100
10
1
0
5000
10000
15000
20000
25000
30000
Genome Position

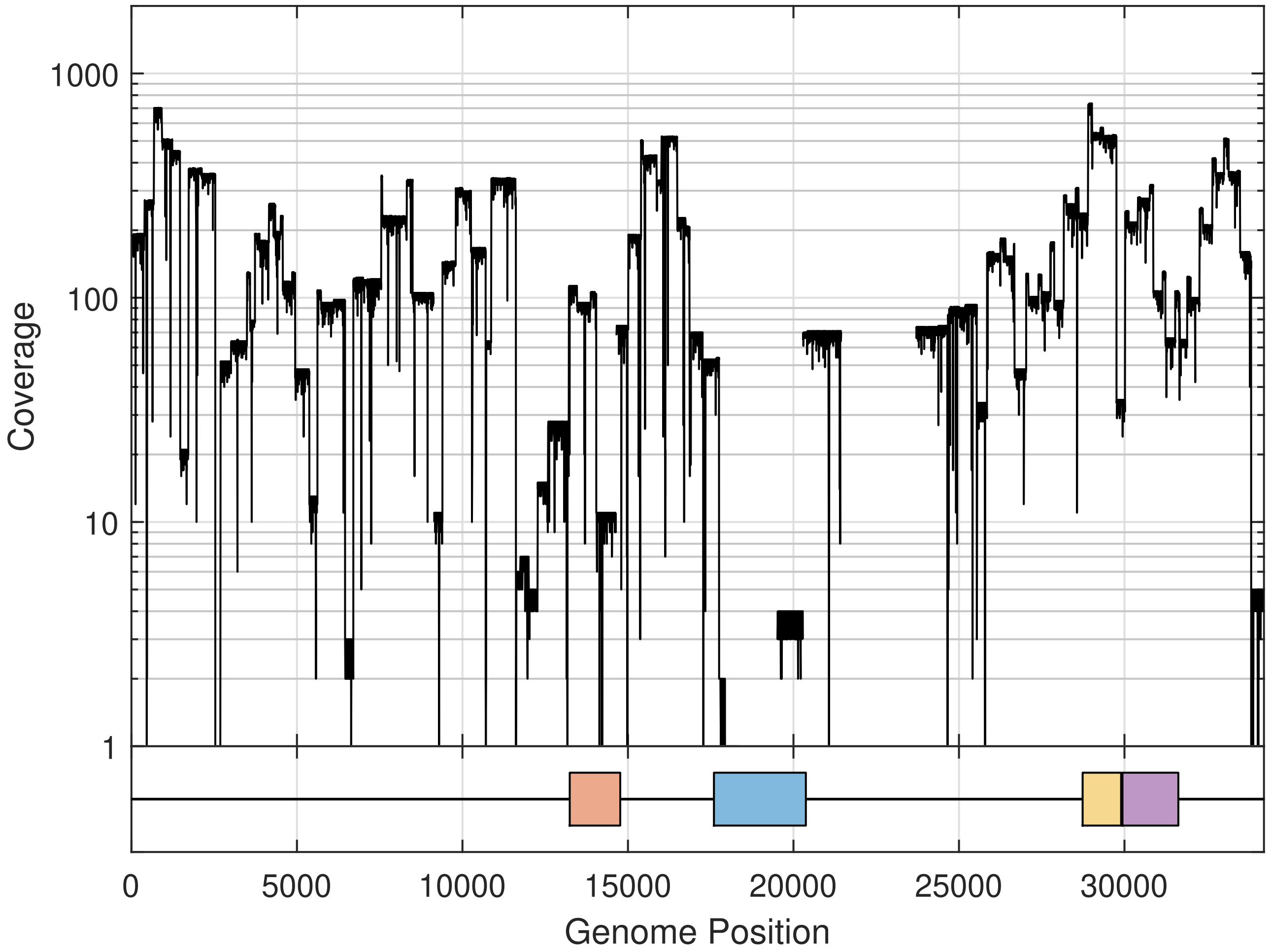

1000
100
10
1
Coverage
0
5000
10000
15000
20000
25000
30000
Genome Position

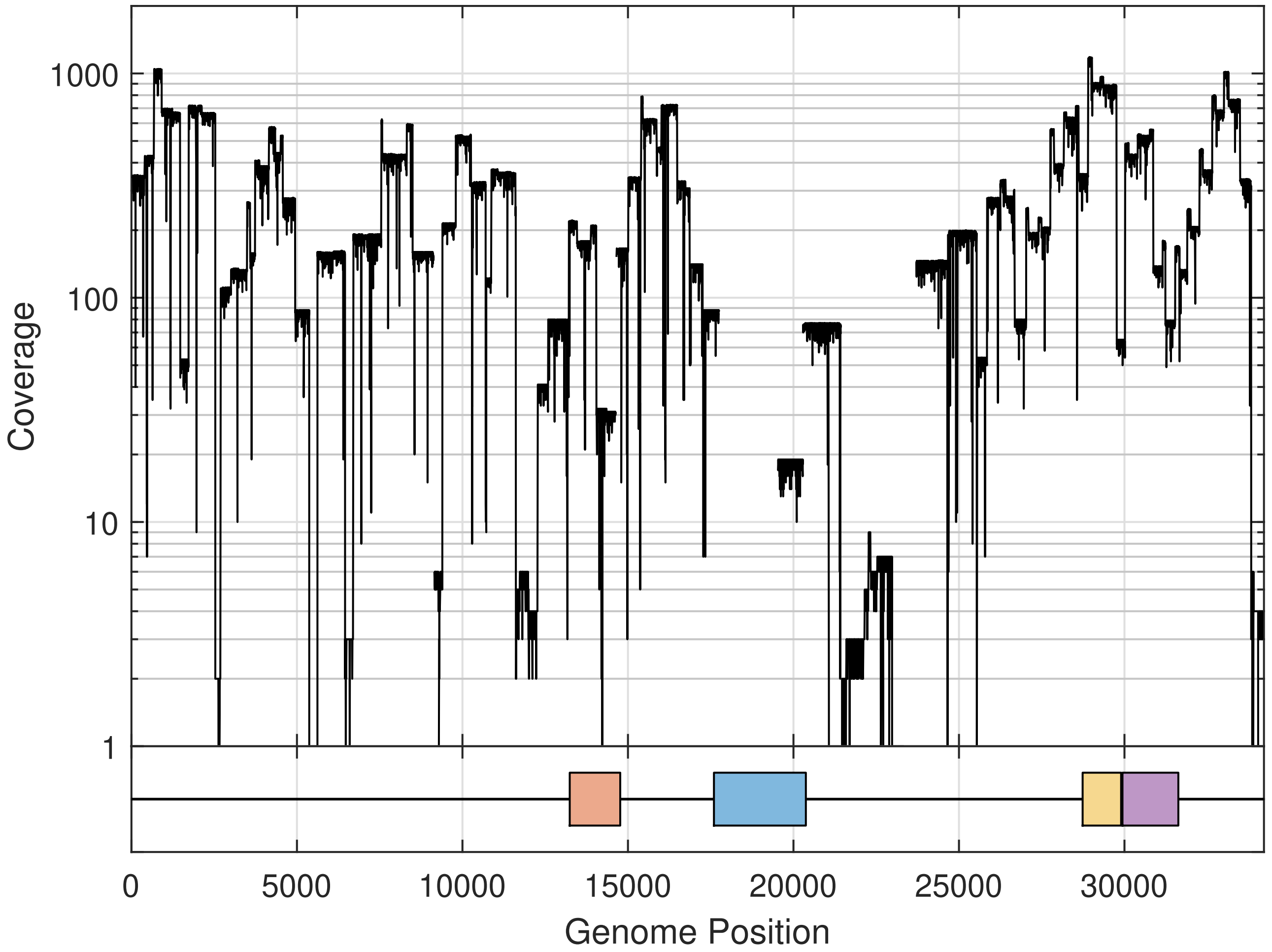

1000
100
10
1
Coverage
0
5000
10000
15000
20000
25000
30000
Genome Position

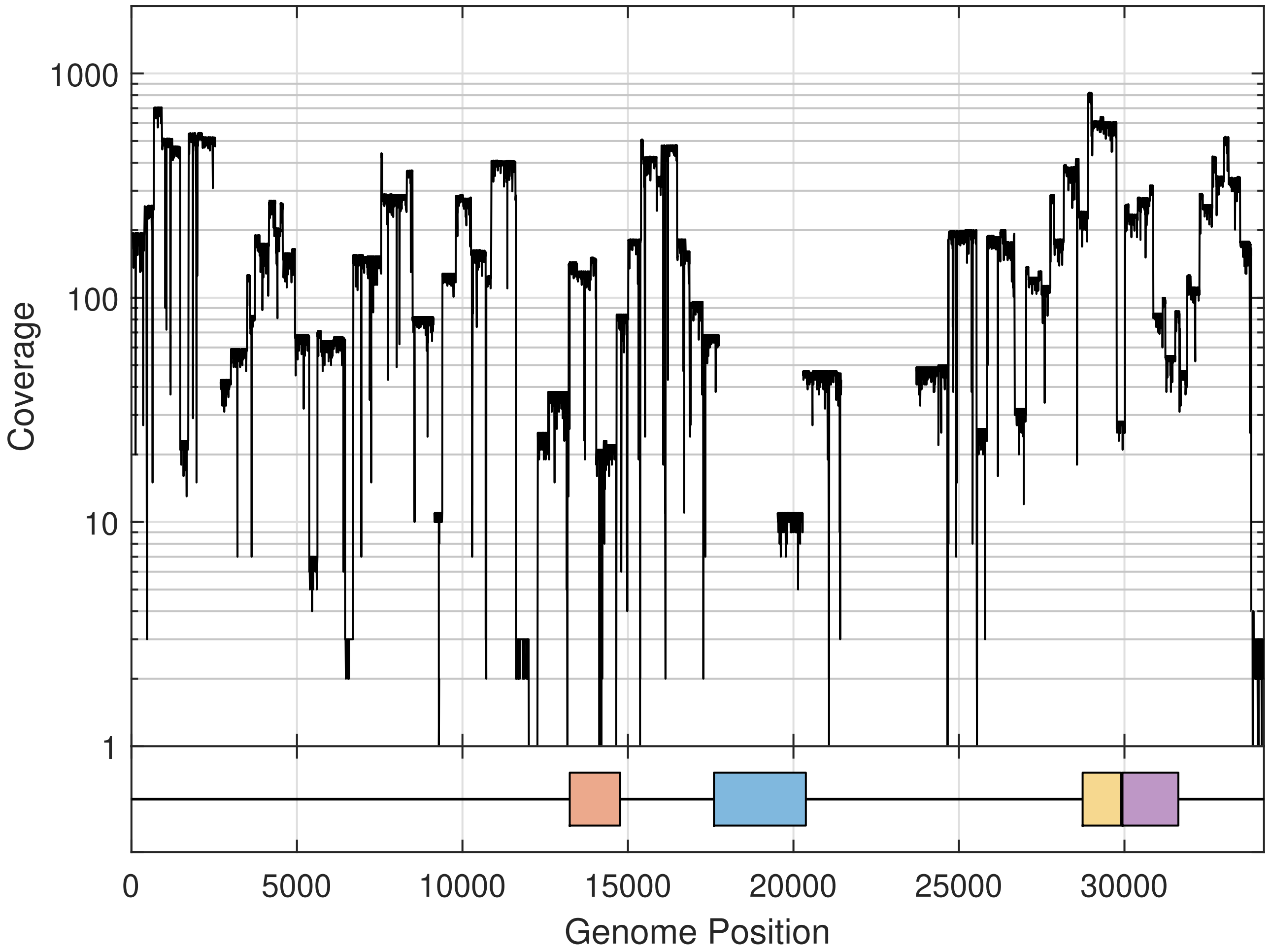

1000
100
10
1
Coverage
0
5000
10000
15000
20000
25000
30000
Genome Position

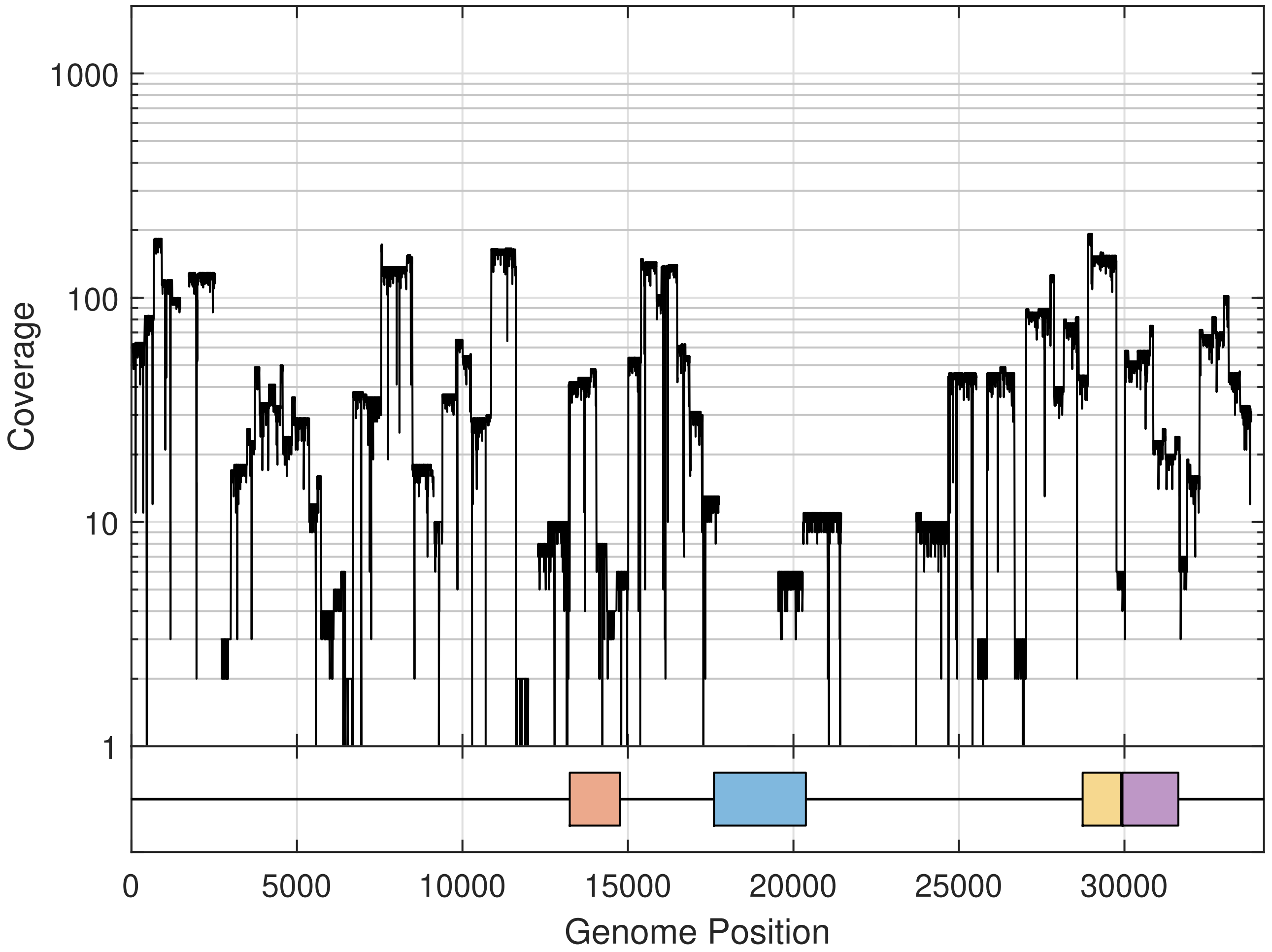

1000
100
10
1
Coverage
0
5000
10000
15000
20000
25000
30000
Genome Position

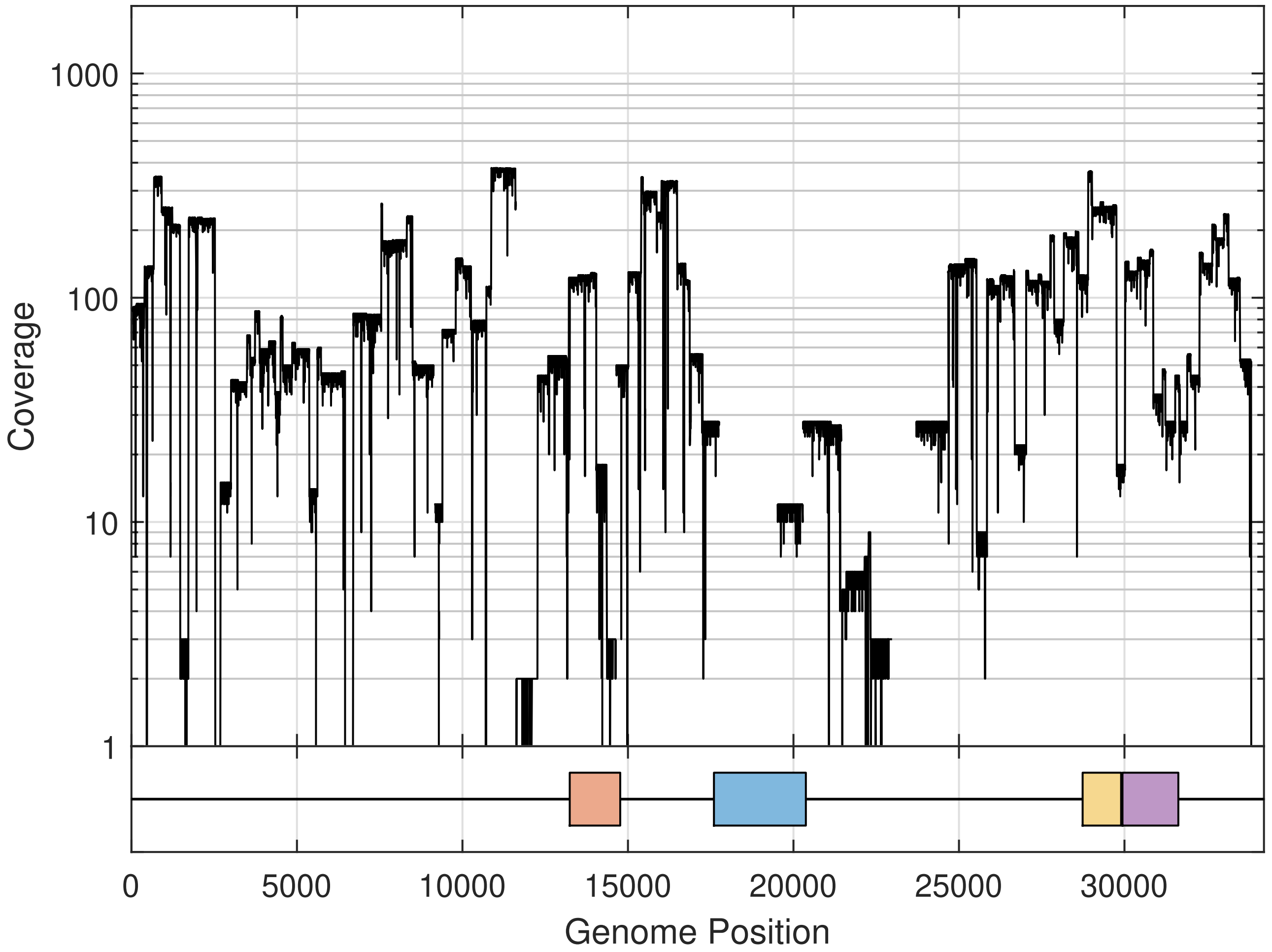

1000
100
10
1
Coverage
0
5000
10000
15000
20000
25000
30000
Genome Position

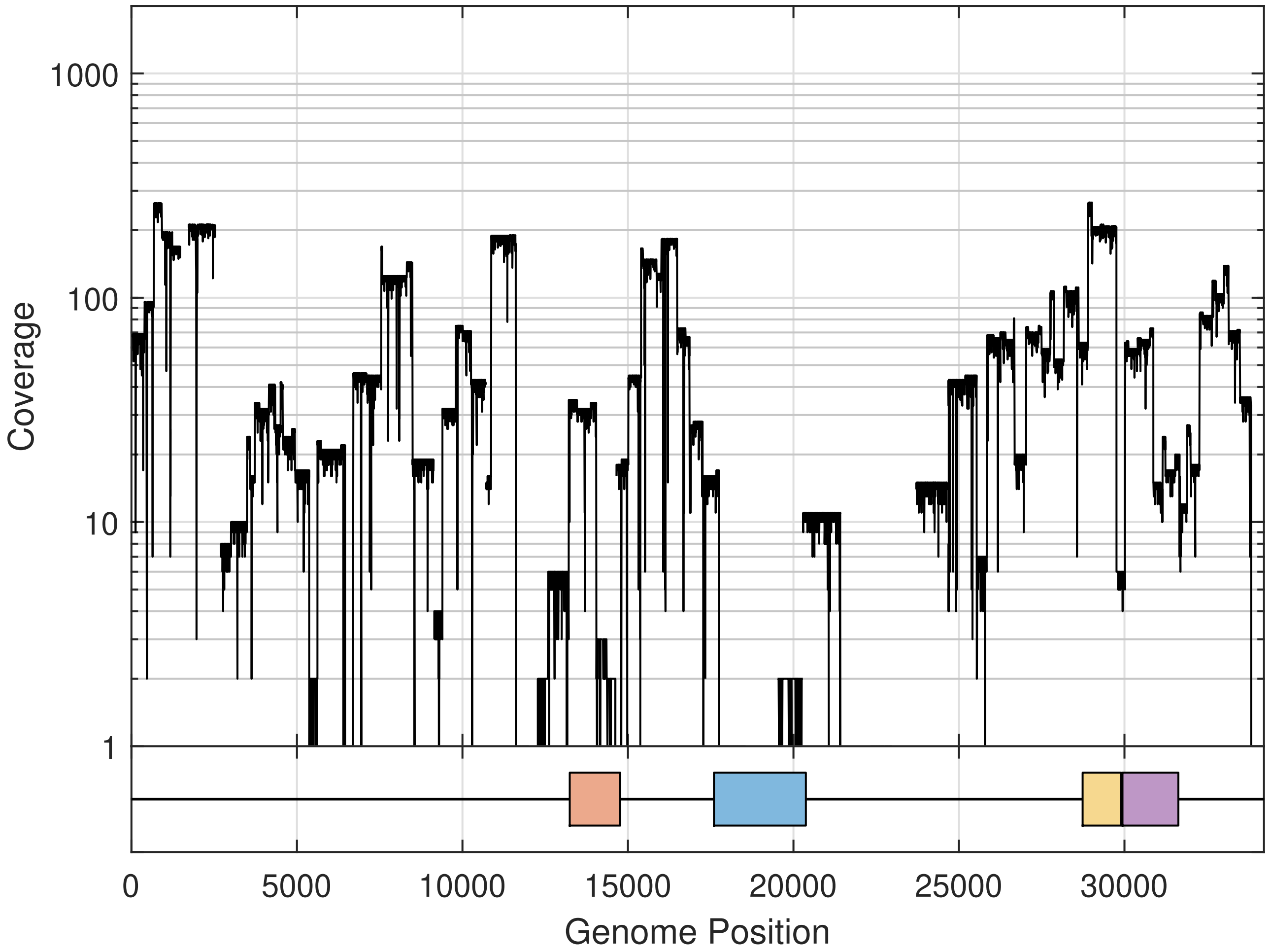

Coverage
1000
100
10
1
0
5000
10000
15000
20000
25000
30000
Genome Position

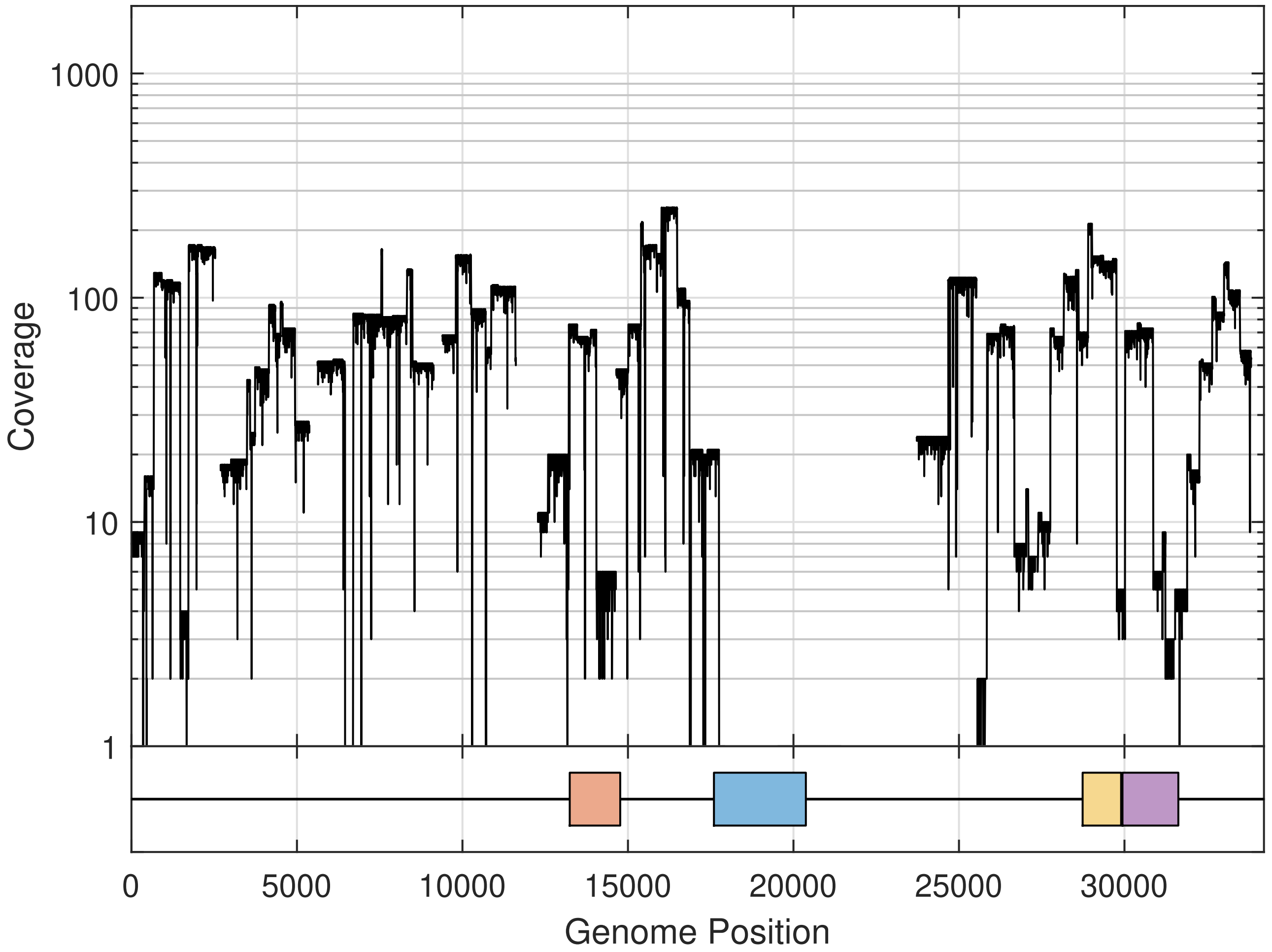

Coverage
1000
100
10
1
0
5000
10000
15000
20000
25000
30000
Genome Position

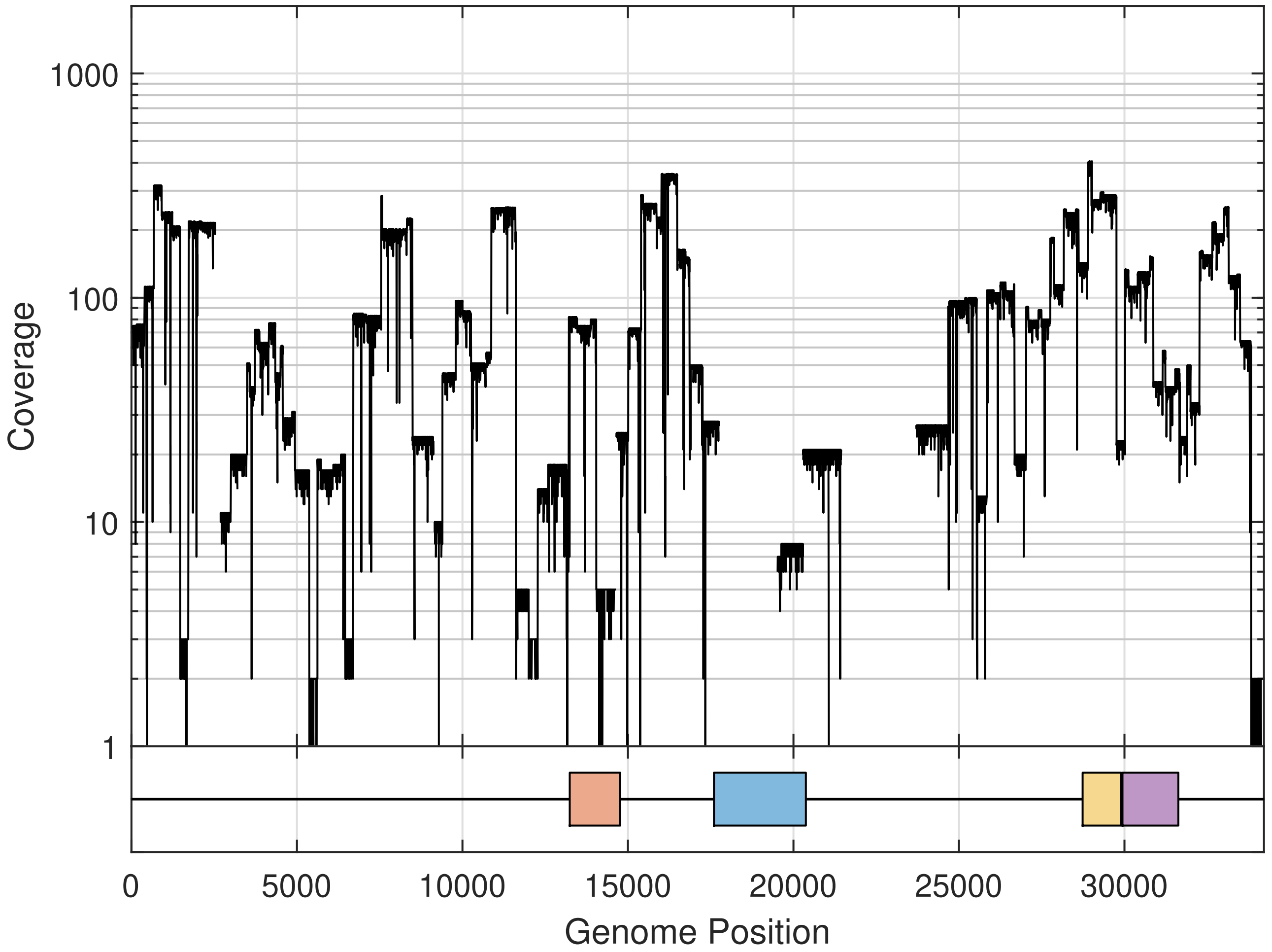
Coverage
1000
100
10
1
0
5000
10000
15000
20000
25000
30000
Genome Position

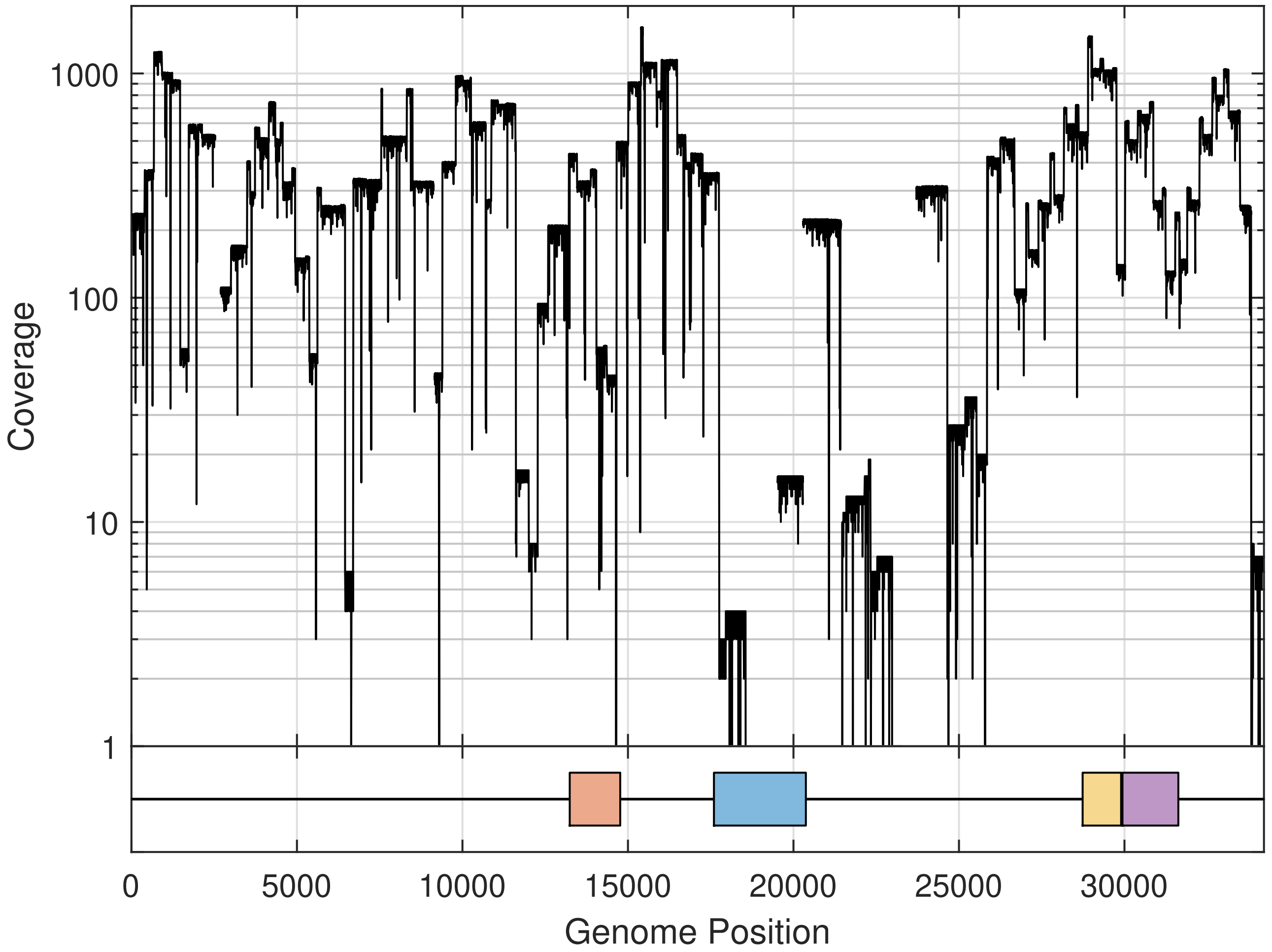

Coverage
1000
100
10
1
0
5000
10000
15000
20000
25000
30000
Genome Position

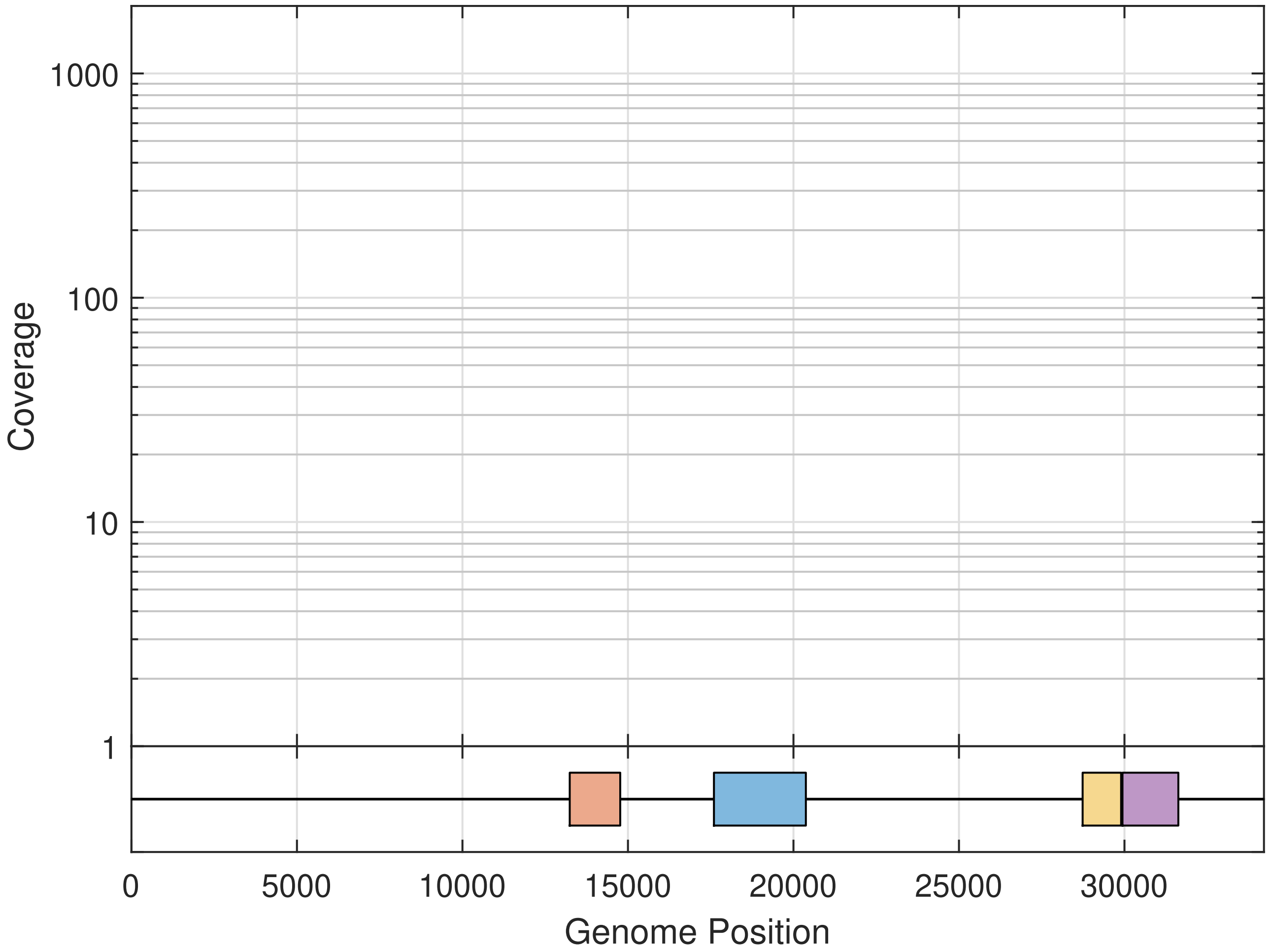

1000
100
10
1
Coverage
0
5000
10000
15000
20000
25000
30000
Genome Position

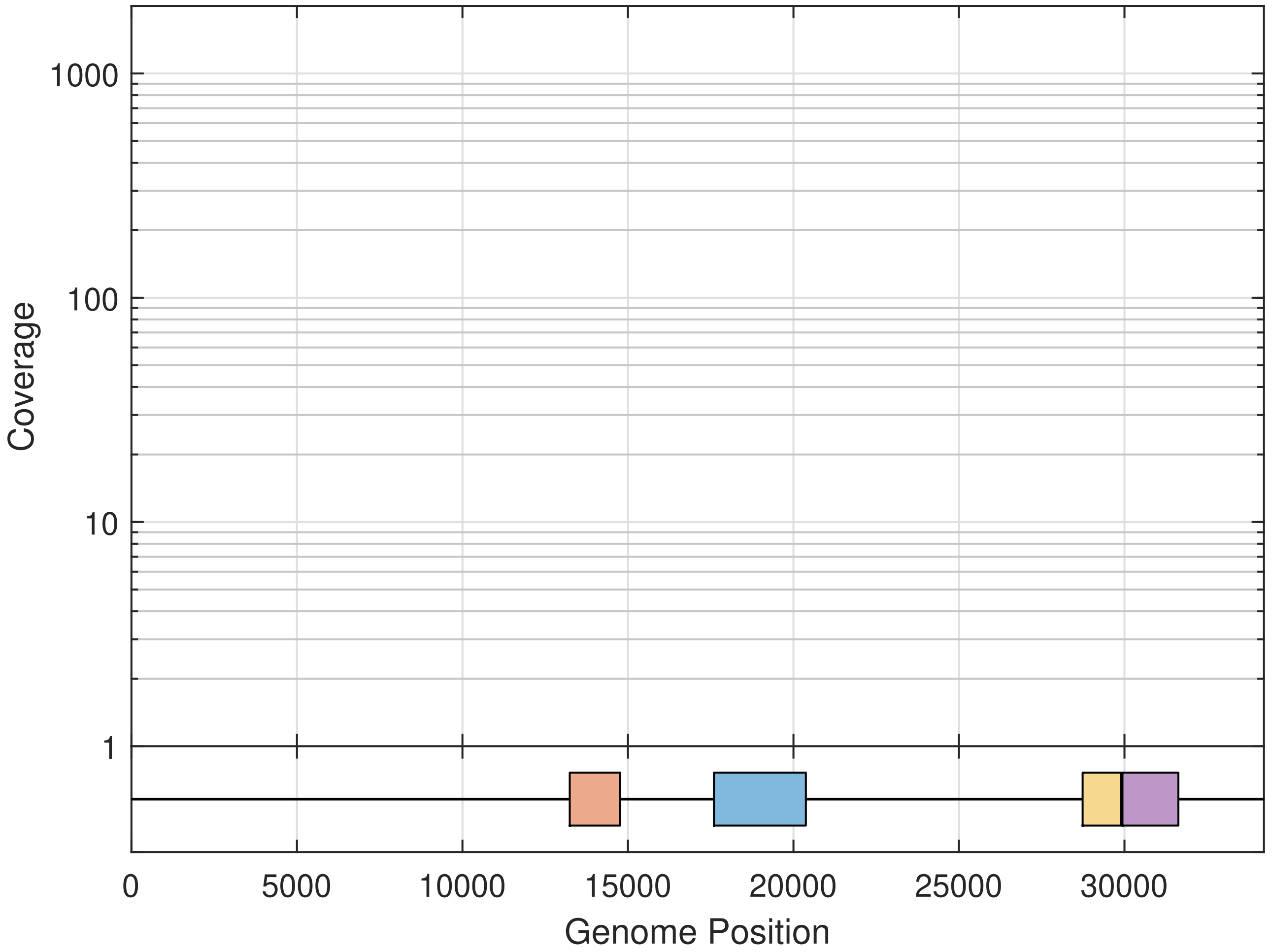

Coverage
1000
100
10
1
0
5000
10000
15000
20000
25000
30000
Genome Position

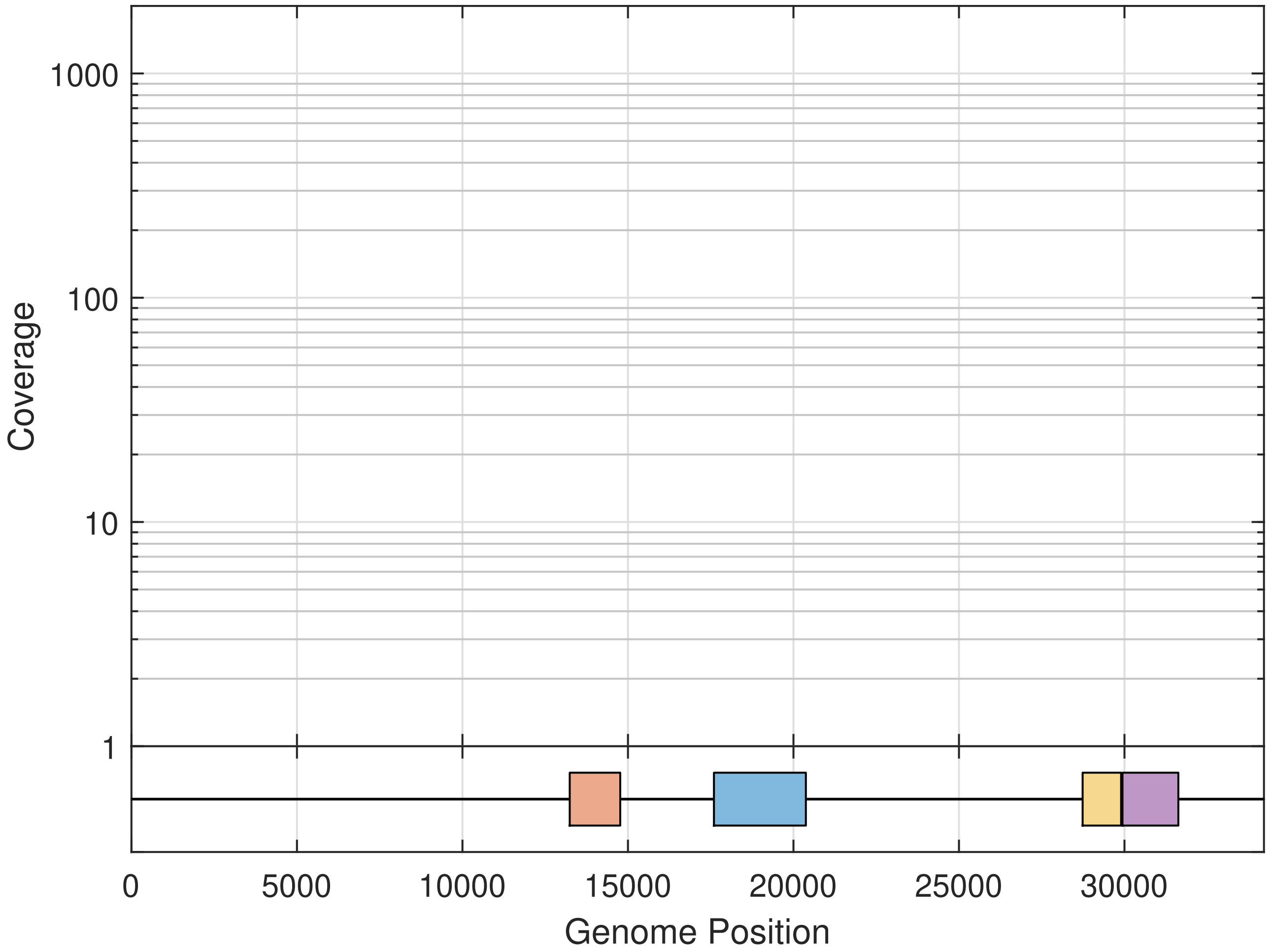

Coverage
1000
100
10
1
0
5000
10000
15000
20000
25000
30000
Genome Position
